# Biological Evaluation of Esters of 4-Carboxylate-1,2,3-triazine and Analogs as New Potential Anti-*Mycobacterium tuberculosis* Agents

**DOI:** 10.3390/molecules31121993

**Published:** 2026-06-07

**Authors:** Gildardo Rivera, Diana V. Navarrete-Carriola, Luca De Angelis, Alma D. Paz-González, Ana Verónica Martínez-Vázquez, Eyra Ortiz-Pérez, Baojie Wan, Scott Franzblau, Marlet Martínez-Archundia, Adriana Moreno-Rodríguez, Isidro Palos, Michael P. Doyle

**Affiliations:** 1 Laboratorio de Biotecnología Farmacéutica, Centro de Biotecnología Genómica, Instituto Politécnico Nacional, Reynosa 88710, Mexico; civky82@gmail.com (D.V.N.-C.); apazg@ipn.mx (A.D.P.-G.); avmartinez@ipn.mx (A.V.M.-V.); eortizp@ipn.mx (E.O.-P.); 2 Department of Chemistry, The University of Texas San Antonio, San Antonio, TX 78249, USA; luca.deangelis@utsa.edu; 3 Institute for Tuberculosis Research, College of Pharmacy, University of Illinois at Chicago, Chicago, IL 60612, USA; baojie@uic.edu (B.W.); sgf@uic.edu (S.F.); 4 Laboratorio de Diseño y Desarrollo de Nuevos Fármacos e Innovación Biotecnológica, Departamento de Posgrado, Escuela Superior de Medicina, Instituto Politécnico Nacional, México City 11340, Mexico; mtmartineza@ipn.mx; 5 Laboratorio de Estudios Epidemiológicos, Clínicos, Diseños Experimentales e Investigación, Facultad de Ciencias Químicas, Universidad Autónoma “Benito Juárez” de Oaxaca, Oaxaca 68120, Mexico; arimor10@hotmail.com; 6 Unidad Académica Multidisciplinaria Reynosa-Rodhe, Universidad Autónoma de Tamaulipas, Reynosa 88779, Mexico; isi_palos@hotmail.com

**Keywords:** antibacterial activity, tuberculosis, triazine derivatives

## Abstract

In searching for novel molecules to act as antibacterial agents, particularly against *Mycobacterium tuberculosis* bacteria, three series of C5- and C6-substituted 1,2,3-triazine compounds were investigated: 1,2,3-triazine-4-carboxylate 1-oxide (series **1**), 1,2,3-triazine-4-carboxylate (series **2**), and 3,6-dihydro-1,2,3-triazine-4-carboxylate 1-oxide derivatives (series **3**). Their structural elucidation was confirmed by ^1^H-NMR, ^13^C-NMR, and HRMS. We determined their antibacterial activity (MIC value) using the MABA against the *M. tuberculosis* H37Rv strain, as well as their physicochemical and pharmacokinetic properties. Finally, to determine their potential mode of action, an inhibition assay against *M. tuberculosis* DNA gyrase was performed. Compounds 4-ethoxycarbonyl-5-(3-methoxyphenyl)-1,2,3-triazine (**2l**) and 4-ethoxycarbonyl-5 -(n-propyl)-1,2,3-triazine (**3s**) exhibited high activity against *M. tuberculosis* with MIC values < 5.90 µg/mL and selectivity index of 18.56 and 8.36, respectively. Additionally, compound **2m** also exhibited anti-mycobacterial activity with MIC values < 10.0 µg/mL. However, none of the selected compounds inhibited the activity of *M. tuberculosis* DNA gyrase, suggesting that another drug target may be involved as a mode of action. These results encourage exploring the use of 1,2,3-triazine as a scaffold for the development of new anti-mycobacterium agents.

## 1. Introduction

*Mycobacterium tuberculosis* is the main causative agent of the chronic infectious disease tuberculosis (TB), an infection with severe complications, and in the worst cases, leading to death [1,2]. Infections caused by *M. tuberculosis* can occur almost anywhere in the body, with respiratory infections being the most common [3]. TB is one of the deadliest diseases worldwide, deeply affecting developing countries. As stated by the World Health Organization (WHO) in 2023, 7.5 million cases of TB were reported in 2022, of which 1.3 million cases resulted in patient death (including patients diagnosed with HIV) [4].

People with TB must take treatment for 6 to 24 months. Long-duration TB treatment increases the probability of developing drug-resistant TB, increasing the cost, complexity of infection, and toxicity exposure caused by the drugs. The current regimen for drug-sensitive cases of TB consists of the use of four first-line drugs (isoniazid, rifampin, pyrazinamide, and ethambutol) administered for a period of 4 to 9 months. When first-line treatment fails, the TB infection becomes resistant (MDR-TB), and a second-line treatment must be administered (2 years of treatment with daily injections for six months). Treatment for MDR-TB tends to fail due to its long duration, cost, toxicity, and side effects of second-line drugs. The main problem with drugs used as first-line treatment is their hepatotoxicity [5]. Therefore, new therapeutic options are necessary and urgent.

DNA gyrases have emerged as a promising pharmacological target in the development of new anti-mycobacterial agents due to their essential role in the replication, transcription, and maintenance of bacterial DNA topology. In *M. tuberculosis*, this enzyme is particularly attractive, as it constitutes the main functional type II topoisomerase, indispensable for the microorganism’s viability and absence in human cells, which favors therapeutic selectivity [6]. In this context, the search for new DNA gyrase inhibitors represents a relevant strategy for the discovery of therapeutic alternatives against resistant strains.

**Figure 1:**
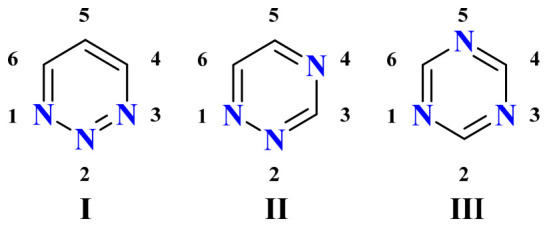
Chemical structure of triazine isomers: (**I**) 1,2,3-triazine; (**II**) 1,2,4-triazine; and (**III**) 1,3,5-triazine. The numbers around the triazine ring represent the position.

Nitrogen-containing heterocycles are important scaffolds in medicinal chemistry and organic chemistry due to their broad applications [7]. Triazine heterocycles are six-membered aromatic compounds that can exist as isomers based on the positions of nitrogen atoms in the benzene ring: 1,2,3-triazine, 1,2,4-triazine, and 1,3,5-triazine (Figure 1) [7,8].

**Figure 2:**
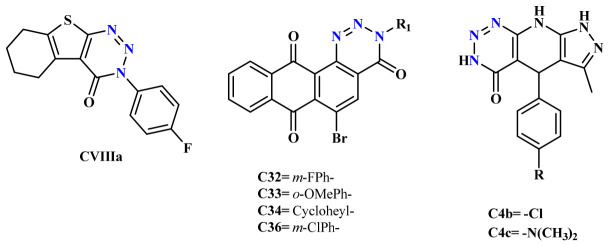
1,2,3-Triazine derivatives with antibacterial activities against Gram-positive and Gram-negative strains.

In general, triazine derivatives have been related to a broad spectrum of biological activities such as antibacterial [9,10], antifungal [9,10], antiprotozoal [11,12], antiviral [13], anticancer [14,15], and neuroprotective agents [16]. So far, only a few reports have exhibited antibacterial activity for 1,2,3-triazine compounds. For example, Saravanan et al. [17] synthesized a series of 3-substituted amino-4,5-tetramethylene thieno [2,3-*d*][1,2,3]-triazine-4(3*H*)-ones with antibacterial activity against Gram-positive and Gram-negative bacterial strains; compound **CVIIIa** (Figure 2) displayed minimum inhibitory concentration (MIC) values in the range of 6.75 to 12.5 µg/mL.

On the other hand, Zvarych’s group reported the synthesis of anthra [1,2-*d*][1,2,3]triazine-4,7,12(3*H*)-triones, which had antistaphylococcal activity with MIC values under 1 µg/mL (**C32**–**C34** and **C36**) [9]. In addition, El-Gohary and coworkers [18] synthesized a series of pyrazolopyridotriazinone (compounds **C4b** and **C4c**) that exhibited good antibacterial activity (Figure 2). Nevertheless, although the antibacterial activity of the triazine derivatives has been investigated, the anti-mycobacterial activity of 1,2,3-triazine compounds is unexplored.

Recently, our research group developed a new method for the synthesis of diverse substituted 1,2,3-triazine derivatives in high yields and under very mild reaction conditions [19,20,21]. Inspired by the potential biological applications and the practical synthetic method for 1,2,3-triazine derivatives, considering the severe side effects caused by drugs used in TB treatment and the increase in antibiotic resistance, this study explores the antibacterial activity of three series of 1,2,3-triazine derivatives against *M. tuberculosis* H37Rv.

## 2. Results

### 2.1. Synthesis

**Figure 3:**
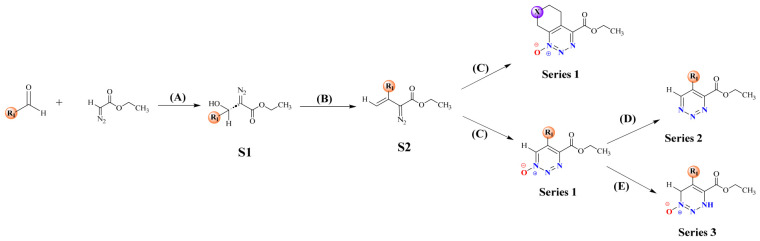
Scheme for the re-synthetic route of 1,2,3-triazine compounds of series **1**, **2**, and **3**. (A) LDA, tetrahydrofuran, −78 °C, 1–2 h; (B) POCl_3_, Et_3_N, CH_2_Cl_2_, 0 °C—rt, 1–24 h; (C) TBN, DCM/HFIPA (20:1), rt, 30 min; (D) Trialkyl phosphite, heat, 60 °C, 6 h; (E) NaBH_4_, TFE, 0 °C, <1 h.

According to the reported procedures [19,20,21], a total of fifty triazine derivatives, grouped in three series (**1**, **2**, and **3**), were re-synthesized and obtained (Figure 3). Initially, 1,2,3-triazine 1-oxides (series **1**) were synthesized via intermolecular [5 + 1] cycloaddition of different vinyl diazoacetate compounds and *tert*-butyl nitrite (TBN) in yields ranging from 51% to 82% [19,21]. 1,2,3-triazines (series **2**) were obtained by deoxygenating the corresponding 1,2,3-triazine 1-oxides using trialkyl phosphites in yields greater than 62% [20]. Also, 3,6-dihydro-1,2,3-triazine 1-oxide (series **3**) was obtained by reduction of the parent 1,2,3-triazine 1-oxides with sodium borohydride (NaBH_4_) in high yields (>73% yield) [19].

This synthetic route provided a set of 1,2,3-triazine compounds with a carboxylate group at C4 and a variety of substituents (alkyl, aryl, and fused heterocyclic systems) at C5 and/or C6 on the triazine scaffold. Series **1** includes nineteen 1,2,3-triazine 1-oxides, series **2** encompasses fifteen 1,2,3-triazines, and series **3** contains sixteen 3,6-dihydro-1,2,3-triazine 1-oxides. All compounds were characterized by proton and carbon nuclear magnetic resonance (^1^H-NMR and ^13^C-NMR) and High-Resolution Mass Spectroscopy (HRMS), and some were structurally confirmed by crystallographic studies (See Supplementary Material, Figure S1). The structural characterization obtained was in accordance with previous reports [19,20,21]. Therefore, these compounds were employed for further biological evaluation studies.

### 2.2. Anti-Mycobacterium Tuberculosis Activity

Since there are no previous reports of anti-mycobacterial activity of 4-carboxylate- 1,2,3-triazine derivatives, series **1**, **2**, and **3** were screened against *M. tuberculosis* H37Rv to determine their MIC values [22]. Results of antimycobacterial activity are reported as MIC_90_ values in µg/mL (Table 1). The MIC values of triazine carboxylates 1-oxide of series 1 were in the range of 100 to 4.62 µg/mL. In general, the results showed that series **2** and **3** had comparable MIC values, but they performed better than series **1**. Compounds **2l**, **3c**, and **3s** had MIC values in the range of 4.62 to 8.72 µg/mL, while **1a**, **1e**, **1h**, **2b**, **3b**, and **3g**, with substitutions at C5 and C6, were less active, with MIC values > 100 µg/mL. The results for these three series of compounds showed that series **2** had MIC values that were reduced by a factor of two to twenty-one times compared with their analogs in series **1**. In the same way, compounds of series **2** and **3** had better MIC values than those of series **1**, except for **2b**, **3b**, and **3g**.

### 2.3. Cytotoxic Evaluation

Cytotoxic activity against the macrophage J774.2 cell line was measured by calculating the half-maximal cytotoxic concentration (CC_50_) for three representative compounds belonging to each triazine series that had MIC values < 100 µg/mL (**1c**, **1j**, **1s**, **2c**, **2j**, **2s**, **3c**, **3j**, and **3s**) (Table 2). 1,2,3-triazine derivatives displayed CC_50_ values in the range of 14.64 to 62.23 µg/mL. However, most of the selective index (SI) values were <2.

### 2.4. ADME Profile Prediction

The physicochemical properties and ADME profiles of the top three triazine derivatives from each series (Table 3) were predicted using the SwissADME server [23].

## 3. Discussion

### 3.1. Anti-Mycobacterial Activity

Initially, all 1,2,3-triazine compounds of series **1**–**3** and the reference drugs (rifampicin and isoniazid) were evaluated in vitro against the growth of *M. tuberculosis* to determine their MIC values (Table 1). Mycobacterial activities were classified as highly active (IC_50_ < 10 µg/mL), moderately active (from 11 to 50 µg/mL), slightly active (IC_50_ from 51 to 99 µg/mL), and inactive (IC_50_ > 100 µg/mL). In series **1**, compounds **1o**–**1q** are the most active, with IC_50_ values of 25 µg/mL. In series **2**, compound **2l** had high anti-mycobacterial activity (IC_50_ = 4.62 µg/mL). In series **3**, compounds **3c** and **3s** were highly active (IC_50_ < 8.72 µg/mL).

#### 3.1.1. Structure–Activity Relationship Analysis Series 1: 1,2,3-Triazine-1-oxide

Nineteen 1,2,3-triazine-1-oxide compounds (series **1**) were obtained using a well-established procedure [19]. Compounds **1a**–**1f**, which contained alkyl groups at the 5-position of the 1,2,3-triazine ring, were also evaluated. Both the length of the carbon chain and the substituent at the end of the chain influenced the anti-mycobacterial activity. The methyl group (**1a**) showed no anti-mycobacterial activity (MIC > 100 µg/mL); the substitution of the chain with an ethyl group (**1b**) and a propyl group (**1c**) had low activity (MIC = 91.00 and 70.10 µg/mL, respectively). Meanwhile, the introduction of a substitution in the chain (**1d**) improved the activity (MIC = 46.60 µg/mL), but the introduction of N_3_ (**1e**) and TBSO (**1f**) terminal groups decreased activity (MIC ≥ 77.26 µg/mL). Furthermore, fused aliphatic rings, such as cyclohexyl (**1g**), and the substitution of oxygen (**1h**) and the NBoc group (**1i**) in the aliphatic ring did not improve activity. These results suggest that the more electronegative the atom in the six-membered ring, the lower the anti-mycobacterial activity.

Compounds with aromatic substitutions at the 5-position on the 1,2,3-triazine ring exhibited greater activity compared to those with alkyl substitutions; compound **1j**, which has a phenyl ring at the 5-position on the triazine ring, showed increased anti-mycobacterial activity (MIC = 48.51 µg/mL). The *para*-methyl substituents (**1k**) and the methoxy group at the *ortho*-position (**1l**) maintained activity (MIC = 47.74 and 48.34 µg/mL, respectively). However, the methoxy group at the *meta*-position (**1m**) drastically reduced the activity (MIC = 90.84 µg/mL). The incorporation of halogens at the *para*-position on the aromatic ring, such as -Cl (**1n**), maintained activity (MIC = 47.63 µg/mL), as did compounds (**1j**–**1l**), while fluorine (**1o**) or bromine (**1p**) and the CF_3_ group (**1q**) at the *para*-position on the aromatic ring increased biological activity (MIC = 25 µg/mL). A similar activity profile was observed when the phenyl ring at the 5-position incorporated fluoride at the 2- and 6- positions (**1r**) and the 2-naphthyl group (**1s**) at the 5-position (MIC = 48.30 and 47.53 µg/mL, respectively), compared to compounds **1j**–**1l** and **1n**.

In summary, aromatic derivatives substituted with halogen atoms (except chlorine) exhibited greater activity; this is potentially due to various factors that may be associated with increased molecular hydrophobicity and, consequently, influence cell wall permeability in mycobacteria; however, further studies are needed that include multiple factors such as logP/logD, polar surface area (PSA), hydrogen bonding capacity, and others, to confirm this association [24,25].

#### 3.1.2. Series 2: 1,2,3-Triazine

In general, the compounds of the 4-carboxylate-1,2,3-triazine series **2** exhibited greater anti-mycobacterial activity (MIC ≤ 100 µg/mL) than their series **1** derivatives. Compounds with aliphatic substitutions with the methyl group (**2a**) exhibited low anti-mycobacterial activity (MIC = 63.7 µg/mL); upon substitution of the chain with an ethyl group (**2b**), activity decreased drastically (MIC > 100 µg/mL); surprisingly, the change to a propyl group (**2c**) and the introduction of a unsaturation in the aliphatic chain (**2d**) and a TBSO terminal group (**2f**) drastically increased the activity (MIC = 16.70, 14.00, and 11.4 µg/mL, respectively). For compounds with the N_3_ terminal group (**2e**) and fused aliphatic rings, such as cyclohexyl (**2g**), and the substitution of oxygen (**2h**) or the NBoc group (**2i**) in the aliphatic ring, no activity was determined.

Compounds with aromatic substituents at the 5-position on the 1,2,3-triazine ring exhibited behavior similar to that of series **1**, where aromatic substitutions exhibit greater activity than aliphatic substitutions. Compounds **2j** and **2k**, with a phenyl ring and a methyl substitution on the phenyl ring at the 5-position on the 1,2,3-triazine ring, exhibited moderate anti-mycobacterial activity (MIC = 11.35 and 11.01 µg/mL, respectively). Meanwhile, the methoxy group at the *meta*-position (**2l**) on the phenyl ring exhibited greater anti-mycobacterial activity (MIC = 4.62 µg/mL), and the methoxy group at the *ortho*-position (**2m**) slightly reduced its activity (MIC = 10.71 µg/mL). Compounds with halogen substitutions at the *para*-position on the phenyl ring, such as -Cl (**2n**), -F (**2o**), -Br (**2p**), and the CF_3_ group (**2q**), exhibited moderate activity (MIC ≥ 12.26 to 46.09 µg/mL). The incorporation of fluoride at the 2- and 6- positions (**2r**) on the phenyl ring and 2-naphthyl (**2s**) at the 5-position on the 1,2,3-triazine ring showed activity similar to that of the compounds with halogens at the *para*-position on the phenyl ring.

Therefore, in series **2**, compound **2l** was identified as the most active compound in the series, corresponding to the derivative with a methoxy group at the *meta*-position on the phenyl ring, demonstrating that the position of the substituent on the aromatic ring plays an important role in its biological activity.

#### 3.1.3. Series 3: 3,6-Dihydro-1,2,3-triazine 1-oxide

The series **3** of 4-carboxylate-3,6-dihydro-1,2,3-triazine 1-oxide derivatives exhibited a wide range of MIC values against *M. tuberculosis*. Compound **3a**, containing a methyl group, exhibited moderate anti-mycobacterial activity (MIC = 47.01 µg/mL); however, extending the aliphatic chain to an ethyl group (**3b**) resulted in no activity against *M. tuberculosis*. Surprisingly, extending the aliphatic chain to three carbons (propyl group) in compound **3c** dramatically increased activity (MIC = 5.09 µg/mL). Meanwhile, introducing unsaturation into the aliphatic chain (**3d**) slightly decreased activity (MIC = 13.60 µg/mL). However, the incorporation of terminal groups in the aliphatic chain, such as N_3_ (**3e**) and TBSO (**3f**) groups, exhibited moderate activity (MIC = 56.08 and 34.90 µg/mL, respectively). For compounds **3g**–**3i**, the activity was not determined.

Compounds with aromatic substituents at the 5-position on the 1,2,3-triazine ring exhibited high and moderate activity. The compound with a phenyl group (**3j**) at the 5-position on the 1,2,3-triazine ring exhibited anti-mycobacterial activity (MIC = 10.47 µg/mL); however, substitutions on the phenyl ring, such as a methyl group at the *para*-position (**3k**) and a methoxy group at the *meta*-position (**3l**), reduced the activity (MIC = 19.84 and 22.50 µg/mL, respectively). Meanwhile, the methoxy group at the *ortho*-position (**3m**) on the phenyl ring exhibited activity similar to compound **3j**. Furthermore, halogen substitutions at the *para*-position on the phenyl ring showed that chlorine (**3n**) and bromine (**3p**) exhibited similar activity (MIC ≥ 19.20 µg/mL), whereas fluorine (**3o**) exhibited greater anti-mycobacterial activity (MIC = 10.50 µg/mL); in contrast, the addition of the CF_3_ group (**3q**) at the *para*-position on the phenyl ring decreased the activity (MIC = 19.20 µg/mL). Finally, the incorporation of fluorine at the 2- and 6- position (**3r**) on the phenyl ring and 2-naphthyl (**3s**) at the 5-position on the 1,2,3-triazine ring showed activity similar to that of compound **3o** (MIC ≤ 10.30 µg/mL).

In summary, series 3 compounds exhibited high activity, such as compound **3c**, which corresponds to aliphatic substitutions, and compound **3s**, which corresponds to aromatic substitutions; therefore, this series appears to be the most promising for the development of new antimycobacterial agents.

### 3.2. Mycobacterium Tuberculosis DNA Gyrase Inhibition Activity Assay

Once the anti-mycobacterial activity of 1,2,3-triazine derivatives was determined, we wondered whether 1,2,3-triazine derivatives could inhibit *M. tuberculosis* DNA gyrase. For this purpose, twelve compounds with MIC < 50 µg/mL were chosen. Four 1,2,3-triazine 1-oxide: **1j**, **1k**, **1l**, and **1o**; Four 1,2,3-triazine: **2j**, **2k**, **2n**, and **2o**; and four 3,6-dihydro-1,2,3-triazine 1-oxide derivatives: **3s**, **3k**, **3o**, and **3n** were selected and examined for inhibition of *M. tuberculosis* DNA gyrase activity (See Supplementary Material, Table S1). None of the 1,2,3-triazine derivatives inhibited *M. tuberculosis* DNA gyrase supercoiling activity at 50 µM. This can be observed in the non-supercoiled DNA in the agarose electrophoresis gel (See Supplementary Material, Figure S2). On the other hand, moxifloxacin (MFX), the positive control, inhibited *M. tuberculosis* DNA gyrase supercoiling activity with an IC_50_ of 6.16 µM (See Supplementary Material, Figures S3 and S4). Based on the results obtained, 1,2,3-triazine derivatives might not bind to *M. tuberculosis* DNA gyrase and therefore not affect its activity; therefore, 1,2,3-triazine derivatives could act by a different mechanism, which is open to further investigation.

### 3.3. ADME Profile Prediction

It is a crucial step in medicinal chemistry that the biological properties of a lead compound must be optimized before that compound can be identified as a drug candidate, which implies the selection of multiple criteria or rules for drug-likeness properties. These rules are strictly related to the physicochemical properties of molecules in question. Nonoptimal physicochemical properties of drug candidates decrease the success rate in the drug development process, which limits a new drug from reaching the market [26]. Lipinski Rule of Five and ADME properties are the first parameters considered in virtual screening or hit molecules improvement. The Lipinski rule encompasses physicochemical properties such as MW ≤ 500, LogP ≤ 5, number of hydrogen bond donors (sum of NH and OH) ≤ 5, and number of hydrogen bond acceptors (sum of N and O) ≤ 10 [27]. In addition, the risk of a drug failing at the clinical trial stage is mediated by Absorption, Distribution, Metabolism, and Excretion (ADME) properties or its pharmacokinetic profile [28].

The ADME properties of the top three triazine derivatives from each series were predicted using the SwissADME server. All compounds exhibit high gastrointestinal (GI) absorption, which favors oral bioavailability, compared to rifampicin, which shows low absorption due to its polarity. Similarly, they are not substrates of P-gp, suggesting a low probability of being expelled by efflux transporters, which favors their intracellular accumulation. In terms of blood–brain barrier (BBB) permeability, most of the triazine derivatives are not permeable, which limits their access to the central nervous system; however, compounds **2c**, **2j**, and **2s** do show BBB permeability, but this does not affect them, as activity in tissues with restricted penetration is not sought.

As for CYP450 isoenzymes, several compounds inhibit CYP1A2, CYP2C19, and CYP2C9, implying a possible risk of metabolic interactions with other drugs that use these pathways. In contrast, inhibition of CYP2D6 and CYP3A4 is generally negative, reducing the likelihood of interactions with major metabolizing enzymes.

All synthesized compounds comply with Lipinski and Veber’s rules, without any violations, indicating favorable properties for oral drugs: adequate molecular weight, moderate lipophilicity, and acceptable molecular flexibility.

The results suggest that the derivatives (series **1**–**3**) have a good oral absorption profile, low brain permeability, and adequate pharmacokinetic compatibility, with potential for oral use and low risk of severe metabolic toxicity. The absence of violations of Lipinski’s and Veber’s rules makes them promising candidates for drug development.

## 4. Materials and Methods

### 4.1. Chemistry

#### 4.1.1. General Conditions

All reactions were performed, unless otherwise stated, in oven-dried (150 °C) glassware with magnetic stirring in an air atmosphere. Reaction progress was followed by analytical thin-layer chromatography (TLC) was performed on silica gel 60 F254 plates (Merck, Darmstadt, Germany) and visualization was accomplished with UV light (254 nm). Column chromatography was performed on CombiFlash^®^ Rf200 (Teledyne Isco, Lincoln, NE, USA) and Rf+ purification systems using normal-phase disposable columns. Melting points were determined uncorrected from an Electro-Thermo Mel-Temp DLX 104 deviced(Electrothermal, Stone, Staffordshire, UK). NMR spectra were recorded on a 500 MHz Bruker Spectrometer (Bruker BioSpin, Rheinstetten, Germany). and calibrated using the resonance signal of the residual undeuterated solvent for ^1^H-NMR [δH = 7.26 ppm (CDCl_3_), δH = 2.54 ppm (CD_3_SOCD_3_)] and deuterated solvent for ^13^C-NMR [δC = 77.16 (CDCl_3_), δC = 39.52 (CD_3_SOCD_3_)] as internal references at 298 K. Spectra were reported as follows: chemical shift (δ ppm), multiplicity (Mi), coupling constants (Hz), integration and assignment. The peak information was described as br = broad, s = singlet, d = doublet, t = triplet, q = quartet, dd = doublet of doublet, m = multiplet, and comp = composite of magnetically non-equivalent protons. ^13^C-NMR spectra were collected on Bruker instruments (126 MHz and 75 MHz) with complete proton decoupling. High-resolution mass spectra (HRMS) were performed on a Bruker MicroTOFESI mass spectrometer with an ESI resource using CsI or LTQ ESI positive ion calibration solution as the standard. Tetrahydrofuran, dichloromethane, chloroform, and toluene were purified using a JC-Meyer solvent purification system. Materials: tert-butyl nitrite (TBN), ethyl diazoacetate (13 wt. % dichloromethane), triethylamine (Et_3_N), phosphoryl chloride (POCl_3_), trimethyl phosphite P(OCH_3_)_3_, triethyl phosphite P(OCH_2_CH_3_)_3_, and sodium borohydride (NaBH_4_), were purchased from TCI, Sigma Aldrich, or Alfa Aesar, and were used without further purification [19,20,21].

#### 4.1.2. Resynthesis of 4-Carboxylate-1,2,3-triazine 1-oxides

Briefly, β-hydroxy diazo compounds used in this work were obtained by a dehydration reaction using POCl_3_ to form a set of nineteen vinyl diazo compounds (**a**–**s**) according to previously reported procedures [19]. The final products (**1a**–**1s**) were synthesized by a [5 + 1] cycloaddition reaction between different vinyl diazo compounds (**a**–**r**) and tert-butyl nitrite, performed under mild conditions at room temperature for 30–60 min using DCM as solvent. The crude product was purified by flash chromatography (% ethyl acetate in hexanes = 20–50%) to give series 1(**1a**–**s**).

#### 4.1.3. Resynthesis of 4-Carboxylate-1,2,3-triazine

The 1,2,3-triazine compounds **2a**–**2d**, **2f**, **2j**–**2s** were synthesized using trialkyl phosphites as deoxygenating agents, as described by Rivera et al., 2022 [20]. Trialkyl phosphite was used as a reactant and the solvent at 60 °C for 6–72 h. Trimethyl phosphite/trimethyl phosphate was removed under reduced pressure, and the residue was purified by flash chromatography (hexane/ethyl acetate, 20–30%) to give the desired series 2.

#### 4.1.4. Resynthesis of 4-Carboxylate-3,6-dihydro-1,2,3-triazine 1-oxides

Following the reported procedure [21], to a solution containing 4-carboxylate-1,2,3-triazine 1-oxide (**1a**–**1g**, **1j**–**2s**), we added sodium borohydride (NaBH_4_) and methanol at 0 °C for 30 min. When the reaction was complete, the solvent was then removed under reduced pressure, and the residue was purified by flash chromatography (hexane/ethyl acetate = 4/1) to give the 3,6-dihydro-1,2,3-triazine 1-oxide compounds of series 3 (**3a**–**3g** and **3j**–**3s**).

### 4.2. Biological Assays

#### 4.2.1. Anti-Mycobacterial Activity: Minimum Inhibitory Concentration

The anti-mycobacterial activity of the 1,2,3-triazine derivatives was assessed in vitro against the *M. tuberculosis* H37Rv ATCC27294 strain according to the modified microplate Alamar blue assay (MABA) [22]. Experiments were performed in duplicate in three independent experiments. The lowest concentration of the assessed compounds that inhibited 90% of *M. tuberculosis* was considered the minimum inhibitory concentration (MIC) value [29].

#### 4.2.2. Determination of Inhibition of *Mycobacterium tuberculosis* DNA Gyrase Supercoiling Assay

The activity of the enzymes was determined before the testing of selected 1,2,3-triazine derivatives, and MFX was used as a positive control. One unit (U) is defined as the amount of the enzyme required for complete supercoiling of the relaxed DNA substrate. All experiments were performed in duplicate. In all assays, the final concentration of DMSO was 1% (*v*/*v*).

Inhibition assay conditions: For the buffer assay, 1 U of *M. tuberculosis* was incubated with 0.5 µg of supercoiled pBR322 DNA in a 30 µL reaction at 37 °C for 30 min under the following conditions: 50 mM HEPES.KOH (pH 7.9), 6 mM magnesium acetate, 4 mM DTT, 1 mM ATP, 100 mM potassium glutamate, 2 mM spermidine, and 0.05 mg/mL albumin. Each reaction was stopped by the addition of 30 µL chloroform/iso-amyl alcohol (24:1) and 30 µL stop dye (40% sucrose (*w*/*v*), 100 mM Tris.HCl (pH 7.5), 10 mM EDTA, 0.5 µg/mL bromophenol blue), before being loaded on a 1.0% TAE gel run at 80 V for 2 h.

Data collection and analysis: Bands were visualized by ethidium staining for 10 min and destaining for 20 min. Gels were scanned using documentation equipment (GeneGenius, Synge, Cambridge, UK), and % inhibition levels (where appropriate) were obtained with gel scanning software using GeneTools software (version M25.1, Syngene, Cambridge, UK).

### 4.3. Physicochemical and Pharmacokinetic Properties ADME

Physicochemical properties and the pharmacokinetic profile of 1,2,3-triazine carboxylates from series 1, 2, and 3, isoniazid, and rifampin were evaluated using the SwissADME server (http://www.swissadme.ch/) (accessed on 23 April 2026) [23]. Briefly, the SMILES (Simplified Molecular Input Line Entry System) of thirteen 4-carboxylate-1,2,3-triazine derivatives (**1j**, **1n**–**1q**, **1s**, **2j**, **2l**, **2m**, **2s**, **3c**, **3j**, and **3s**) were used to obtain predictions [27,28].

### 4.4. Cytotoxicity

The mouse macrophage from the J774.2 cell line (donated by Unidad de Investigacion Medica del Centro Medico Nacional Siglo XXI, Mexico) was maintained in culture flasks with RPMI 1640 medium supplemented with 10% FBS, 100 U µg/mL penicillin, 100 mg/mL streptomycin, and glutamine (2 mM) at 37 °C and in a 5% CO_2_ atmosphere. The medium was replaced at 2–3-days intervals. The cells were incubated with different concentrations of the 4-carboxylate-1,2,3-triazine derivatives **1j**, **1n**–**1q**, **1s**, **2j**, **2l**, **2m**, **2s**, **3c**, **3j**, and **3s** (200–0.80 µM), incubated for 48 h at 37 °C and 5% CO_2_ atmosphere. Cells in the presence of the maximum concentration of DMSO (0.2%) were included as a negative control; the metabolic activity of the cells was determined using the MTT method. The percentage of cell viability was calculated, and the half-maximal cytotoxic concentration (CC_50_) was determined by Probit analysis. Three independent assays were performed in triplicate each [30,31].

## 5. Conclusions

In this work, three series of 1,2,3-triazine derivatives were resynthesized and tested for their anti-mycobacterial activities. 1,2,3-triazines (series **2**) and 3,6-dihydro-triazines 1-oxide (series **3**) showed better anti-mycobacterium activity than their analogous 1,2,3-triazines 1-oxides (series **1**). The most active compounds in series **1** were compounds **1o**, **1p**, and **1q** (MIC = 25 µg/mL), which contain halogens such as -F and -Br and the -CF_3_ group in the *para*-position on the phenyl ring at the 5-position on the 1,2,3-triazine ring; for series **2**, it was compound **2l** (MIC = 4.62 µg/mL) with a methoxy group at the *meta*-position on the phenyl ring at the 5-position on the 1,2,3-triazine ring; and for series 3, compound **3c** with a propyl group and compound **3s** with a 2-naphthyl group (MIC = 5.9 µg/mL and 8.72 µg/mL, respectively). Although these activity values were not comparable to those of the reference drugs rifampicin (MIC = 0.09 µg/mL) and isoniazid (MIC = 0.25 µg/mL), they are proposed as potential scaffolds for developing new anti-mycobacterial agents.

Furthermore, since this is the first study of 4-carboxylate-1,2,3-triazine derivatives against *M. tuberculosis*, it is necessary to continue the search for the pharmacological target that will help determine the possible mechanism of action of these compounds.

## Figures and Tables

**Table 1:** Antimycobacterial activity of 1,2,3-triazine carboxylate series 1, 2, and 3 against *M. tuberculosis* H37Rv strain (MIC in µg/mL).

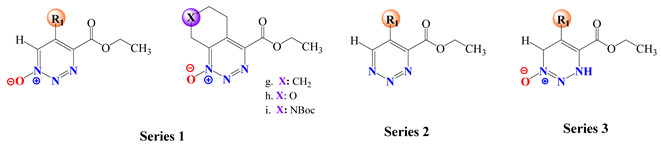					
Code	R1	X	MIC_90_ (µg/mL)		
			Series		
			1	2	3
a	CH_3_-	-	>100	63.7	47.01
b	CH_3_-CH_2_-	-	91.00	>100	>100
c	CH_3_CH_2_CH_2_-	-	70.10	16.70	5.90
d	*cis* CH_3_-CH=CH_2_-CH_2_-	-	46.60	14.00	13.60
e	N_3_CH_2_CH_2_-	-	>100	ND	56.08
f	OTBS-CH_2_CH_2_-	-	77.26	11.4	34.90
g	cyclohexyl-	CH_2_	84.99	ND	ND
h	cyclohexyl	O	>100	ND	ND
i	cyclohexyl	NBoc	93.92	ND	ND
j	C_6_H_5_-	-	48.51	11.35	10.47
k	*p*-CH_3_C_6_H_5_-	-	47.74	11.01	19.84
l	*m*-CH_3_OC_6_H_4_-	-	48.34	4.62	22.50
m	*o*-CH_3_OC_6_H_4_-	-	90.84	10.71	11.13
n	*p*-ClC_6_H_4_-	-	47.63	19.42	24.42
o	*p*-FC_6_H_4_-	-	25.00	12.26	10.50
p	*p*-BrC_6_H_4_-	-	25.00	36.35	24.10
q	*p*-CF_3_C_6_H_4_-	-	25.00	46.09	19.20
r	2,6-F_2_C_6_H_3_-	-	48.30	17.30	10.30
s	2-C_10_H_7_-	-	47.53	23.47	8.72
RMP			0.09		
INH			0.25		

ND = not determined; RMP = rifampicin; INH = isoniazid, MIC = minimum inhibitory concentration.

**Table 2:** Half-maximal cytotoxic activity (CC_50_) and selectivity index (SI) of nine 1,2,3-triazine compounds against macrophage J774.2.

Compound	CC_50_ (µg/mL)	MIC_90_ (µg/mL)	SI
**1c**	>52.65	70.10	>0.75
**1j**	>49.11	48.51	>1.01
**1s**	32.85	47.53	0.69
**2c**	62.23	16.70	3.72
**2j**	22.44	11.35	1.98
**2s**	55.86	23.47	2.38
**3c**	14.64	5.9	2.48
**3j**	49.45	10.47	4.72
**3s**	21.69	8.72	2.49

**Table 3:** Pharmacokinetic profile of selected 1,2,3-triazine compounds determined by SwissADME.

ID	GI Absorption	BBB Permeant	Pgp Substrate	CYP1A2 Inhibitor	CYP2C19 Inhibitor	CYP2C9 Inhibitor	CYP2D6 Inhibitor	CYP3A4 Inhibitor	Lipinski No. Violations	Veber No. Violations
**1c**	High	No	No	Yes	No	No	No	No	0	0
**1j**	High	No	No	Yes	No	No	No	No	0	0
**1s**	High	No	No	Yes	Yes	No	No	No	0	0
**2c**	High	Yes	No	Yes	Yes	No	No	No	0	0
**2j**	High	Yes	No	Yes	Yes	No	No	No	0	0
**2s**	High	Yes	No	Yes	Yes	No	No	No	0	0
**3c**	High	No	No	Yes	No	No	No	No	0	0
**3j**	High	No	No	No	No	No	No	No	0	0
**3s**	High	No	No	No	No	No	No	No	0	0

GI = gastrointestinal absorption; BBB = blood–brain barrier; Pgp = permeability glycoprotein.

## Data Availability

The data presented here, and the Supporting Information, can be openly shared upon request to the corresponding author: giriveras@ipn.mx.

## Supplementary Materials

The following supporting information can be downloaded at https://www.mdpi.com/article/10.3390/molecules31121993/s1, Figure S1. ORTEP drawing of 3j (ethyl 1-oxo-5-phenyl-3,6-dihydro-1,2,3λ^5^-triazine-4-carboxylate); Table S1: Inhibition of *M. tuberculosis* DNA Gyrase activity; Figure S2. Inhibition *M. tuberculosis* DNA gyrase supercoiling activity by 1,2,3-triazine derivatives at 50 µM. (1) Assay 1; (2) Assay 2; Figure S3. Determination of the IC_50_ of moxifloxacin as a control. (A) Assay 1 (6.16 µM); (B) Assay 2 (5.12 µM); Figure S4. Percentage of supercoiling activity by moxifloxacin. (A) Assay 1 (6.16 µM); (B) Assay 2 (5.12 µM).

## Abbreviations

*M. tuberculosis*	*Mycobacterium tuberculosis*
TB	Tuberculosis
MDR-TB	Multidrug-resistant TB
EDG	Electron-donating groups
