# Supplementary material for: Biological Evaluation of Esters of 4-Carboxylate-1,2,3-triazine and Analogs as New Potential Anti-Mycobacterium tuberculosis Agents
Source: Molecules. 2026 Jun 7;31(12):1993. doi: 10.3390/molecules31121993 (PMC13304873; doi:10.3390/molecules31121993)
Supplement: Supplementary file 1 [file molecules-31-01993-s001.zip › molecules-4339577-supplementary.pdf]

# Supporting information

# Biological Evaluation of Esters of 1,2,3-triazine-4-carboxylate and Analogs as New Potential Anti-*Mycobacterium Tuberculosis* Agents

Gildardo Rivera[1*], Diana V. Navarrete-Carriola[1,2], Luca De Angelis[2], Alma D. Paz-Gonzalez[1], Ana Vernica Martinez-Vazquez[1], Eyra Ortiz-Perez[2], Baojie Wan[3], Scott Franzblau[3], Marlet Martinez-Archundia[4], Adriana Moreno-Rodriguez[5], Isidro Palos[6], Michael P. Doyle[2*]

[1] *Laboratorio de Biotecnología Farmacéutica, Centro de Biotecnología Genómica, Instituto Politécnico Nacional, 88710 Reynosa, México*
[2] *Department of Chemistry, The University of Texas San Antonio, San Antonio, Texas 78249, United States*
[3] *Institute for Tuberculosis Research, College of Pharmacy, University of Illinois at Chicago, Chicago, IL 60612, USA.*
[4] *Laboratorio de Diseño y Desarrollo de Nuevos Fármacos e Innovación Biotecnológica, Departamento de Posgrado, Escuela Superior de Medicina, Instituto Politécnico Nacional, México City, México*
[5] *Laboratorio de Estudios Epidemiológicos, Clínicos, Diseños Experimentales e Investigación, Facultad de Ciencias Químicas, Universidad Autónoma "Benito Juárez" de Oaxaca, Oaxaca 68120, México*
[6] *Unidad Académica Multidisciplinaria Reynosa-Rodhe, Universidad Autónoma de Tamaulipas, Reynosa 88779, Mexico*

***Corresponding autor**

| | |
|---|---|
| Gildardo Rivera | Michael Doyle |
| Laboratorio de Biotecnología Farmacéutica | Deparment of Chemistry |
| Centro de Biotecnología Genómica | Biotechnology Sciences and Engineering Building |
| Instituto Politécnico Nacional | University of Texas at San Antonio |
| Reynosa, 88710, México | One UTSA Circle San Antonio, 78249, Texas |


## CONTENTS

1. **Crystallographic Data for Compound 3j** ........ 2
2. **Structural elucidation** ........ 2
3. **NMR spectra** ........ 6
4. ***M. tuberculosis* gyrase supercoiling inhibition** ........ 18

## 1. Crystallographic Data for Compound 3j

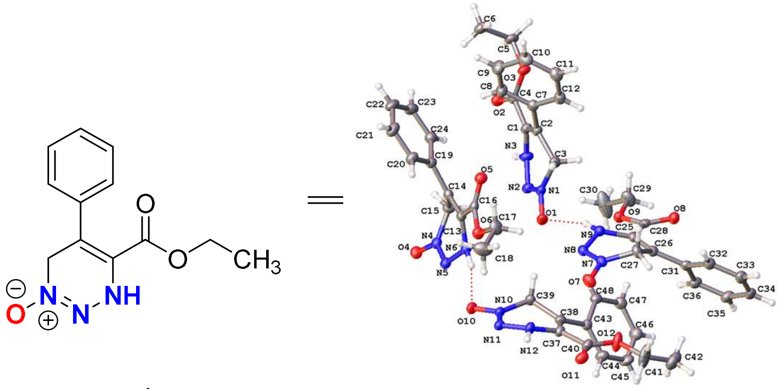

3j

CCDC: 2088139

**Figure S1.** ORTEP drawing of **3j** (ethyl 1-oxo-5-phenyl-3,6-dihydro-1,2,3$\lambda^5$-triazine-4-carboxylate)

## 2. Structural elucidation

### 2.1 Series 1

The spectroscopic data of **1a**, **1f**, **1g**, **1h, 1i**, **1j**, **1k**, **1s**, **1l**, **1m**, **1o**, **1n**, and **1q** were identical to those previously reported by De Angelis et al., 2021 [a].

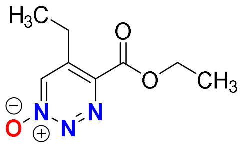

**5-ethyl-4-Ethoxycarbonyl-1,2,3-triazine 1-Oxide, 1b.** Orange oil, yield: 85 %. **$^{1}$H NMR** (500 MHz, $CDCl_3$) δ 7.99 (s, 1H), 4.52 (q, *J* = 7.4 Hz, 1H), 3.03 (q, *J* = 7.5 Hz, 2H), 1.48 (t, *J* = 7.1 Hz, 2H), 1.34 (t, *J* = 7.4 Hz, 2H). **$^{13}$C NMR** (126 MHz, $CDCl_3$) δ 162.20, 143.12, 137.04, 134.69, 62.89, 23.44, 14.15, 12.74. **HRMS** (ESI) calculated for $[M+H]^+$: $C_8H_{11}N_3O_3$ m/z: 198.0873, observed: 198.0868.

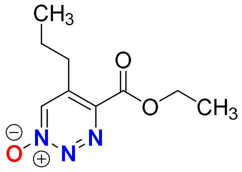

**5-(*n*-propyl)-4-Ethoxycarbonyl-1,2,3-triazine 1-Oxide**, **1c.** orange oil, yield: 72 %. **$^{1}$H NMR** (500 MHz, $CDCl_3$) δ 7.96 (s, 1H), 4.52 (q, *J* = 6.9 Hz, 2H), 2.94 (t, 2H), 1.67 (tq, *J* = 7.7, 7.5 Hz, 2H), 1.48 (t, 3H), 0.93 (t, *J* = 6.9 Hz, 3H). **$^{13}$C NMR** (126 MHz, $CDCl_3$) δ 162.22, 142.11, 135.05, 62.88, 31.45, 30.02, 28.84, 22.26, 14.15, 13.83. **HRMS** (ESI) calculated for $[M+H]^+$: $C_9H_{13}N_3O_3$ m/z: 212.1030, observed: 212.1025.

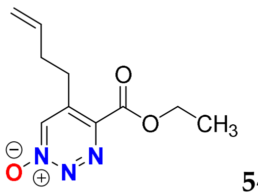

**5-(But-3-en-1-yl)-4-ethoxycarbonyl-1,2,3-triazine 1-Oxide, 1d**. Orange oil, yield: 62 %. **$^{1}$H NMR** (500 MHz, $CDCl_3$) δ 7.94 (s, 1H), 5.80 (ddt, *J* = 16.9, 10.1, 6.6 Hz, 1H), 5.14 – 5.05 (m, 2H), 4.53 (q, *J* = 7.2 Hz, 2H), 3.07 (t, *J* = 7.5 Hz, 2H), 2.46 (q, *J* = 7.2 Hz, 2H), 1.48 (t, *J* = 7.1 Hz, 3H). **$^{13}$C NMR** (126 MHz, $CDCl_3$) δ 141.03, 137.04, 135.25, 135.00, 117.70, 62.94, 32.64, 29.33, 14.15. **HRMS** (ESI) calculated for $[M+H]^+$: $C_{10}H_{13}N_3O_3$ m/z: 224.1030, observed: 224.1025.

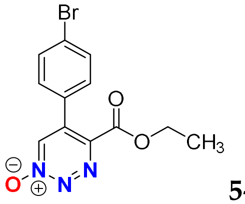

**5-(4-Bromophenyl)-4-ethoxycarbonyl-1,2,3-triazine 1-Oxide, 1p.** White solid. mp: 138.0-140.6 °C, yield: 86 %. **$^{1}$H NMR** (500 MHz, CDCl3) δ 8.02 (s, 1H), 7.71 (d, J = 8.7 Hz, 2H), 7.30 (d, J = 8.5 Hz, 2H), 4.39 (q, J = 7.2 Hz, 2H), 1.31 (t, J = 7.1 Hz, 3H). **$^{13}$C NMR** (126 MHz, CDCl3) δ 162.06, 137.65, 133.83, 132.62, 129.59, 129.02, 126.09, 63.16, 13.90. **HRMS** (ESI) calculated for $[M+H]^+$: $C_{12}H_{10}BrN_3O_3$ m/z: 323.9978, observed: 323.9974.

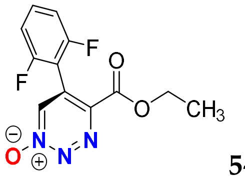

**5-(2,6-Difluorophenyl)-4-ethoxycarbonyl-1,2,3-triazine 1-Oxide**, **1r**: White solid m.p.: 153.4-155 °C, yield: 76 %. **$^{1}$H NMR** (500 MHz, $CDCl_3$) δ 8.07 (s, 1H), 7.60 – 7.53 (m, 1H), 7.12 (t, *J* = 9.2 Hz, 2H), 4.41 (q, *J* = 7.1 Hz, 2H), 1.33 (t, *J* = 7.1 Hz, 3H). **$^{13}$C NMR** (126 MHz, $CDCl_3$) δ 161.31, 160.33, 158.37, 135.76, 133.26 (t, *J* = 10.2 Hz), 128.26, 112.13 (dd, *J* = 20.4, 4.1 Hz), 63.05, 13.86. **HRMS** (ESI) calculated for $[M+H]^+$: $C_{12}H_9F_2N_3O_3$ m/z: 282.0685, observed: 282.0680.

### 2.2 Series 2

The spectroscopic data of **2a, 2b, 2c, 2f, 2j, 2k, 2l, 2m, 2n, 2o, 2p,** and **2q** were identical to those previously reported by Rivera et al., 2022 [b].

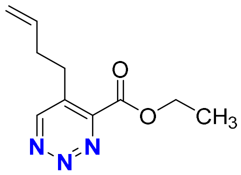

**ethyl 5-(but-3-en-1-yl)-1,2,3-triazine-4-carboxylate, 2d.** Yellow oil, yield: 62%. **$^{1}$H NMR** (500 MHz, $CDCl_3$) δ 9.05 (s, 1H), 5.86 – 5.76 (m, 1H), 5.10 – 5.00 (m, 2H), 4.57 (q, *J* = 7.2 Hz, 2H), 3.05 (t, *J* = 7.5 Hz, 2H), 2.47 (q, *J* = 7.9 Hz, 2H), 1.50 (t, *J* = 7.2 Hz, 3H). **$^{13}$C NMR** (126 MHz, $CDCl_3$) δ 163.45, 152.67, 149.70, 135.24, 132.07, 117.43, 63.15, 33.26, 28.85, 14.13. **HRMS** (ESI) calculated for $[M+H]^+$: $C_{10}H_{13}N_3O_2$ m/z: 208.1081, observed: 208.1081.

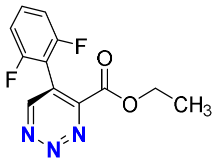

**ethyl 5-(2,6-difluorophenyl)-1,2,3-triazine-4-carboxylate, 2r.** White solid mp: 99.7-100.8 °C, yield: 75 %. **$^{1}$H NMR** (500 MHz, $CDCl_3$) δ 9.26 (s, 1H), 7.56 (ddd, *J* = 15.0, 8.5, 6.4 Hz, 1H), 7.13 (t, *J* = 8.2 Hz, 2H), 4.47 (q, *J* = 7.1 Hz, 2H), 1.36 (t, *J* = 7.2 Hz, 3H). **$^{13}$C NMR** (126 MHz, $CDCl_3$) δ 162.71, 160.65, 160.60, 158.65, 158.60, 152.13, 148.90, 132.96, 132.88, 132.80, 120.45, 112.23, 112.20, 112.07, 112.04, 63.29, 13.85. **HRMS** (ESI) calculated for $[M+H]^+$: $C_{12}H_9F_2N_3O_2$ m/z: 266.0736, observed: 266.0736.

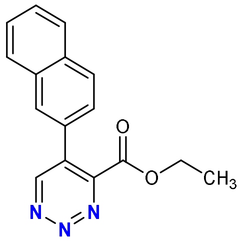

**ethyl 5-(naphthalen-2-yl)-1,2,3-triazine-4-carboxylate, 2s.** Brown solid,mp: 126.0-127.9 °C, yield 82 %. **$^{1}$H NMR** (500 MHz, $CDCl_3$) δ 9.35 (s, 1H), 8.03 (d, *J* = 12.5 Hz, 2H), 7.95 (d, *J* = 8.1 Hz, 2H), 7.68 – 7.61 (m, 2H), 7.54 (d, *J* = 8.4 Hz, 1H), 4.41 (q, *J* = 7.1 Hz, 2H), 1.24 (t, *J* = 7.1 Hz, 3H). **$^{13}$C NMR** (126 MHz, $CDCl_3$) δ 163.80, 150.61, 150.20, 133.83, 133.07, 129.48, 129.32, 128.87, 128.52, 128.33, 128.13, 127.95, 127.49, 124.77, 63.19, 13.86. **HRMS** (ESI) calculated for $[M+H]^+$: $C_{16}H_{13}N_3O_3$ m/z: 280.1081, observed: 280.1075.

## 2.3 Series 3

The spectroscopic data of **3a, 3c, 3e, 3f, 3j, 3k, 3l, 3m, 3n, 3o, 3q** and **3s**were identical to those previously reported by De Angelis et al., 2023 [c].

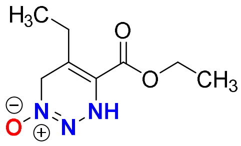

**4-(ethoxycarbonyl)-5-ethyl-3,6-dihydro-1,2,3-triazine 1-oxide, 3b.** Yellow oil, yield: 92 %. **$^1$H NMR** (500 MHz, $CDCl_3$) δ 8.39 (s, 1H), 4.51 (s, 2H), 4.39 (q, 2H), 2.67 (q, *J* = 7.5 Hz, 2H), 1.41 (t, *J* = 7.1 Hz, 3H), 1.14 (t, *J* = 7.5 Hz, 3H). **$^{13}$C NMR** (126 MHz, $CDCl_3$) δ 160.67, 123.69, 120.36, 62.96, 62.40, 23.27, 14.13, 12.35. **HRMS** (ESI) calculated for $[M+H]^+$: $C_8H_{13}N_3O_3$ m/z: 200.1030, observed: 200.1024.

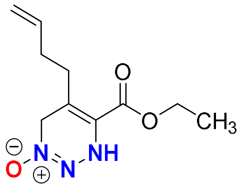

**5-(but-3-en-1-yl)-4-(ethoxycarbonyl)-3,6-dihydro-1,2,3-triazine 1-oxide, 3d.** Yellow solid, mp:61.8-62.9 °C, yield 85 %. **$^1$H NMR** (500 MHz, $CDCl_3$) δ 8.42 (s, 1H), 5.80 (ddt, *J* = 16.9, 10.2, 6.7 Hz, 1H), 5.10 – 5.02 (m, 2H), 4.48 (s, 2H), 4.40 (q, *J* = 7.1 Hz, 2H), 2.76 (t, *J* = 7.4 Hz, 2H), 2.29 (q, *J* = 7.2 Hz, 2H), 1.41 (t, *J* = 7.2 Hz, 3H). **$^{13}$C NMR** (126 MHz, $CDCl_3$) δ 160.65, 136.40, 124.66, 118.23, 116.47, 63.61, 62.46, 32.28, 29.58, 14.13. **HRMS** (ESI) calculated for $[M+H]^+$: $C_{10}H_{15}N_3O_3$ m/z: 226.1186, observed: 226.1186.

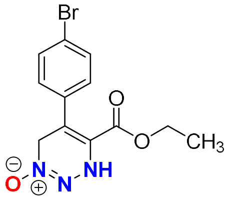

**5-(4-bromophenyl)-4-(ethoxycarbonyl)-3,6-dihydro-1,2,3-triazine 1-oxide, 3p.** Yellow solid, mp: 130.9-133.1 °C, yield: 80 %. **$^1$H NMR** (500 MHz, $CDCl_3$) δ 8.80 (s, 1H), 7.53 (d, *J* = 8.5 Hz, 2H), 7.15 (d, *J* = 8.5 Hz, 2H), 4.75 (s, 2H), 4.22 (q, *J* = 7.1 Hz, 2H), 1.15 (t, *J* = 7.2 Hz, 3H). **$^{13}$C NMR** (126 MHz, $CDCl_3$) δ 160.43, 132.88, 131.45, 130.61, 125.17, 123.30, 112.82, 64.17, 62.66, 13.70. **HRMS** (ESI) calculated for $[M+H]^+$: $C_{12}H_{12}BrN_3O_3$ m/z: 326.0135, observed: 326.0134.

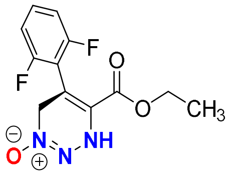

**5-(2,6-difluorophenyl)-4-(ethoxycarbonyl)-3,6-dihydro-1,2,3-triazine 1-oxide, 3r.** White solid, mp: 99.7-100.8 °C, yield: 83 %. **$^1$H NMR** (500 MHz, $CDCl_3$) δ 9.26 (s, 1H), 7.56 (ddd, *J* = 15.0, 8.5, 6.4 Hz, 1H), 7.13 (t, *J* = 8.2 Hz, 2H), 4.47 (q, *J* = 7.1 Hz, 2H), 1.36 (t, *J* = 7.2 Hz, 3H). **$^{13}$C NMR** (126 MHz, $CDCl_3$) δ 162.71, 160.65, 160.60, 158.65, 158.60, 152.13, 148.90, 132.96, 132.88, 132.80, 120.45, 112.23, 112.20, 112.07, 112.04, 63.29, 13.85. **HRMS** (ESI) calculated for $[M+H]^+$: $C_{12}H_{11}F_2N_3O_3$ m/z: 284.0841, observed: 284.0841.

## 3. NMR spectra

## Series 1

**¹H-NMR** (500 MHz, $CDCl_3$) of compound 1b

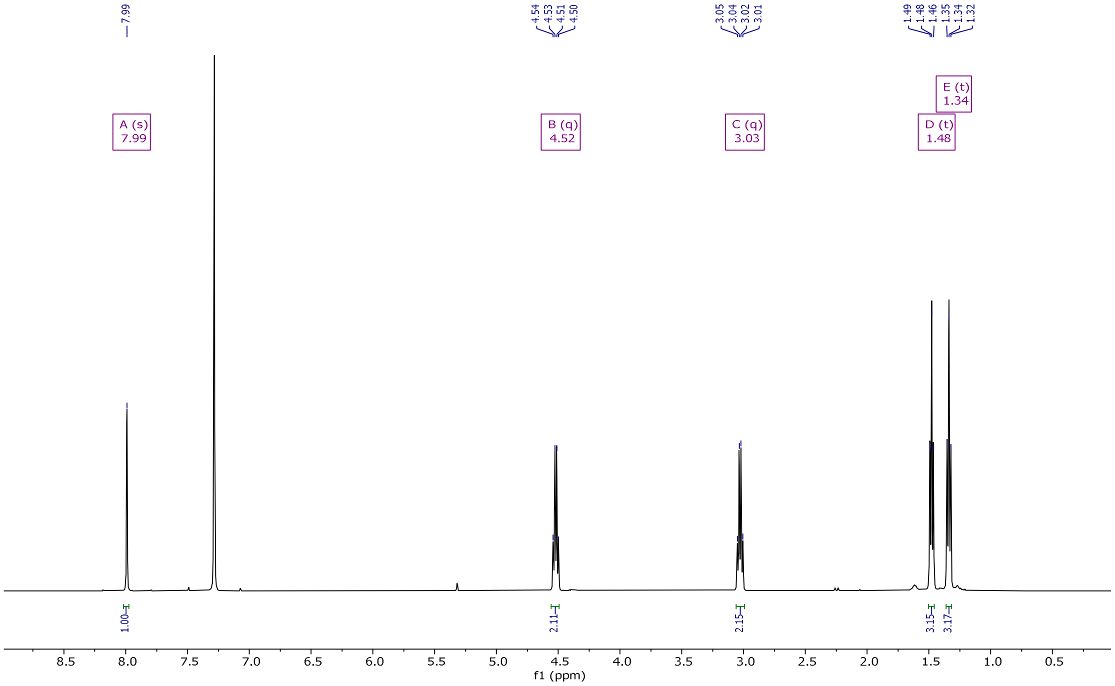


**¹³C-NMR** (126 MHz, $CDCl_3$) of compound 1b

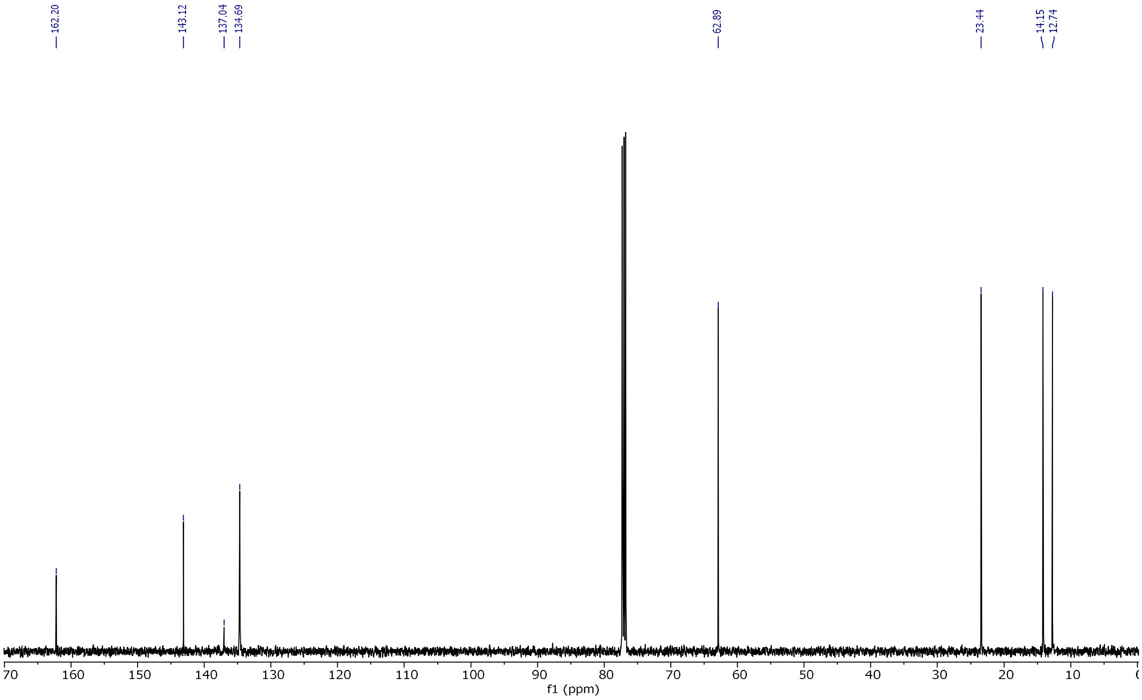

**$^{1}$H-NMR** (500 MHz, $CDCl_3$) of compound 1c

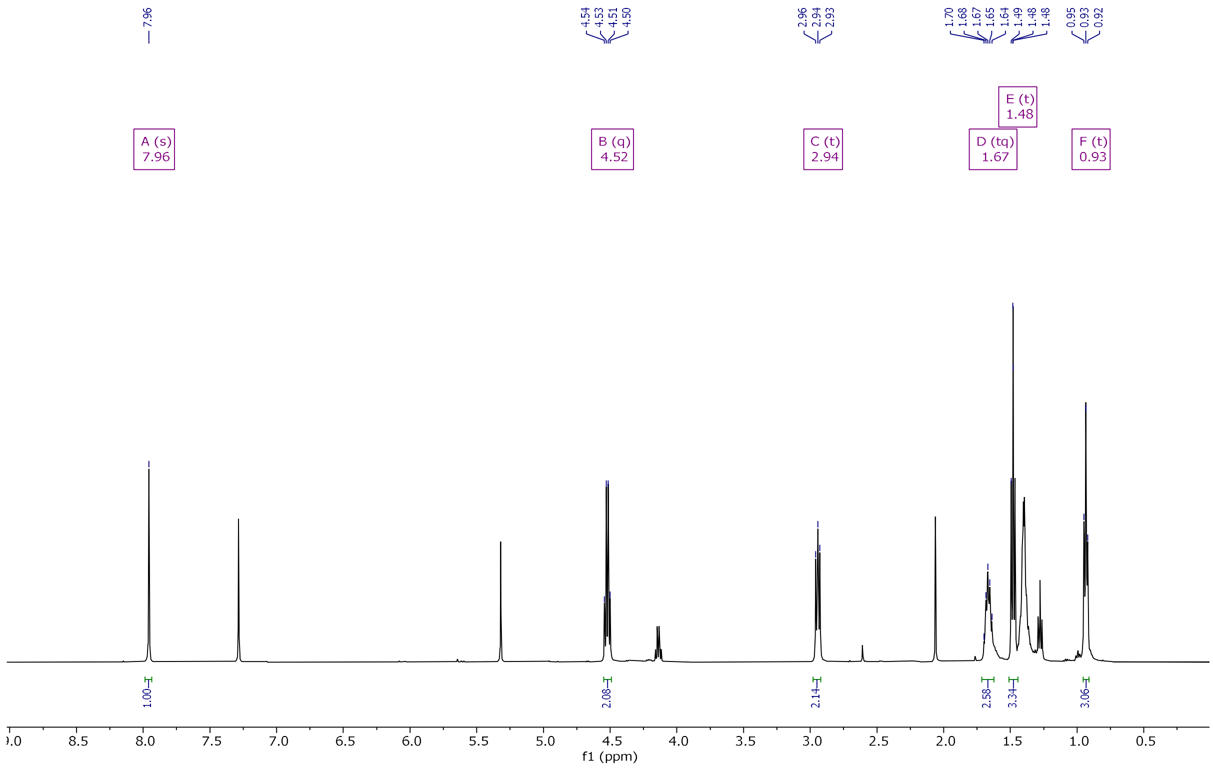


**$^{13}$C-NMR** (126 MHz, $CDCl_3$) of compound 1c

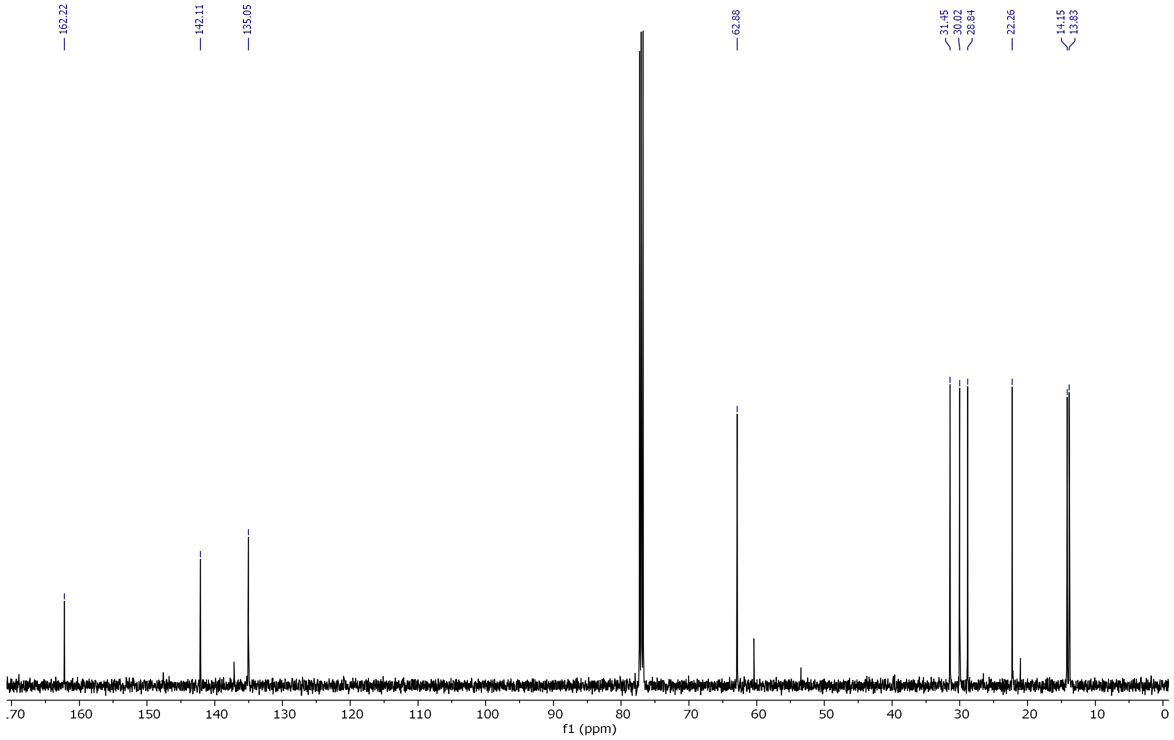

**$^{1}$H-NMR** (500 MHz, $CDCl_3$) of compound 1d

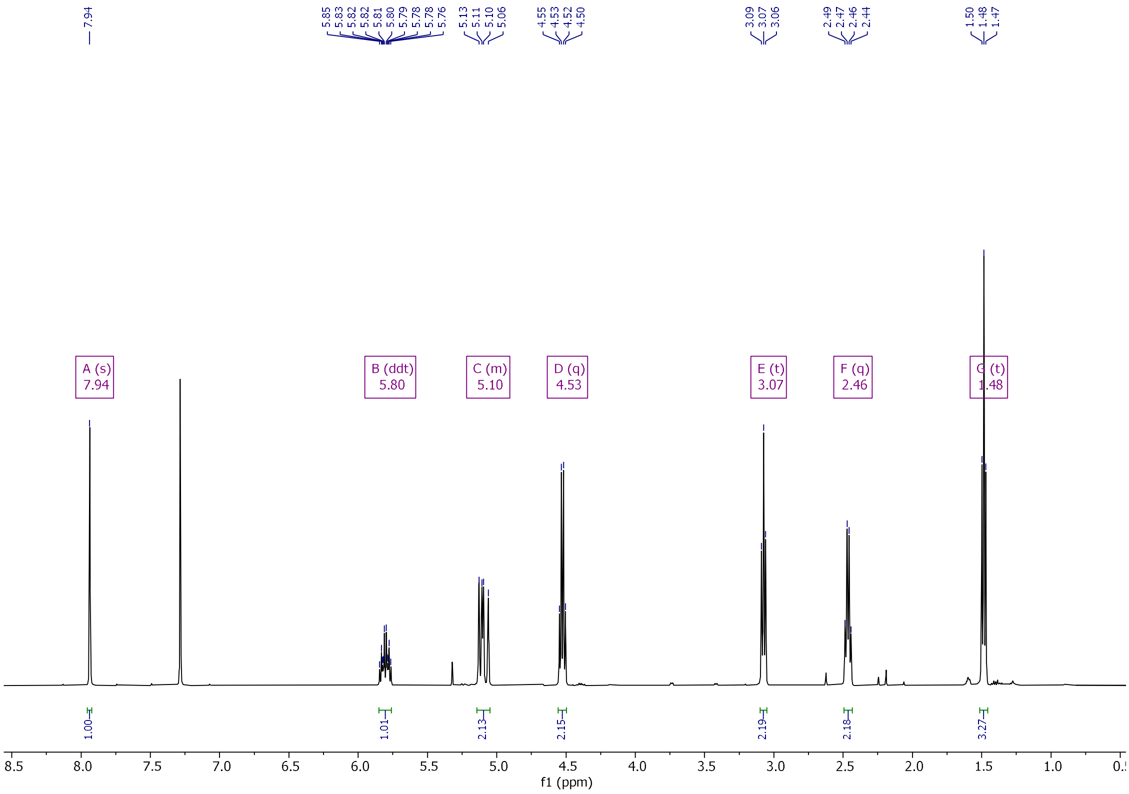


**$^{13}$C-NMR** (126 MHz, $CDCl_3$) of compound 1d

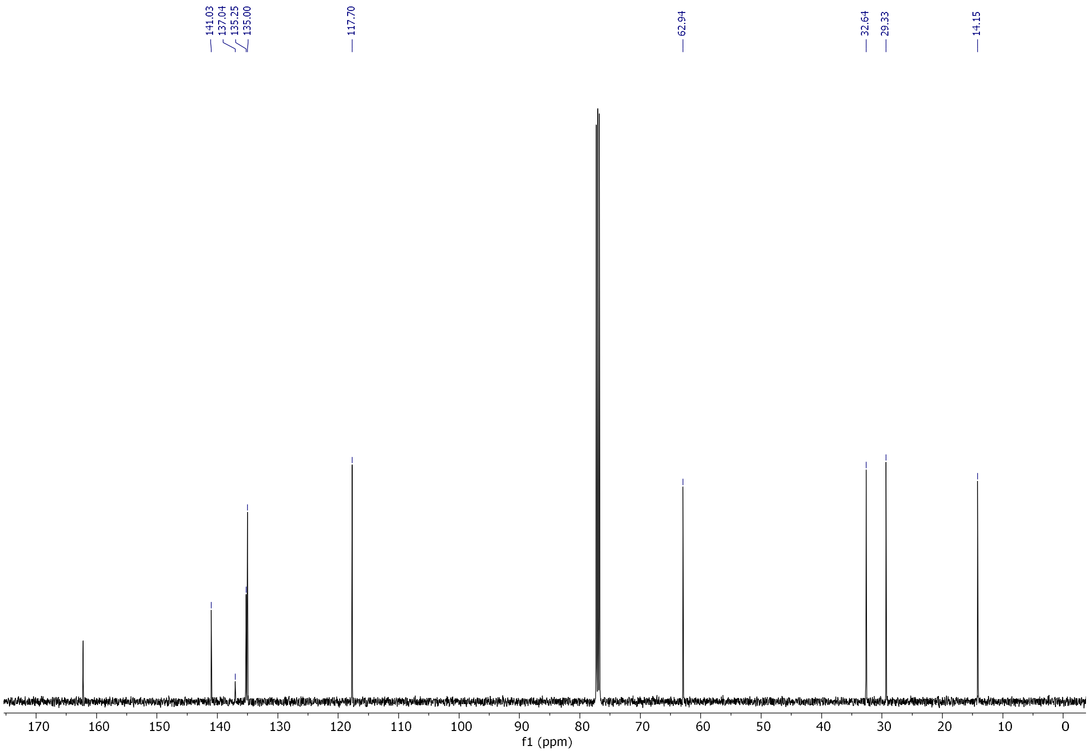

**$^{1}$H-NMR** (500 MHz, $CDCl_3$) of compound 1p

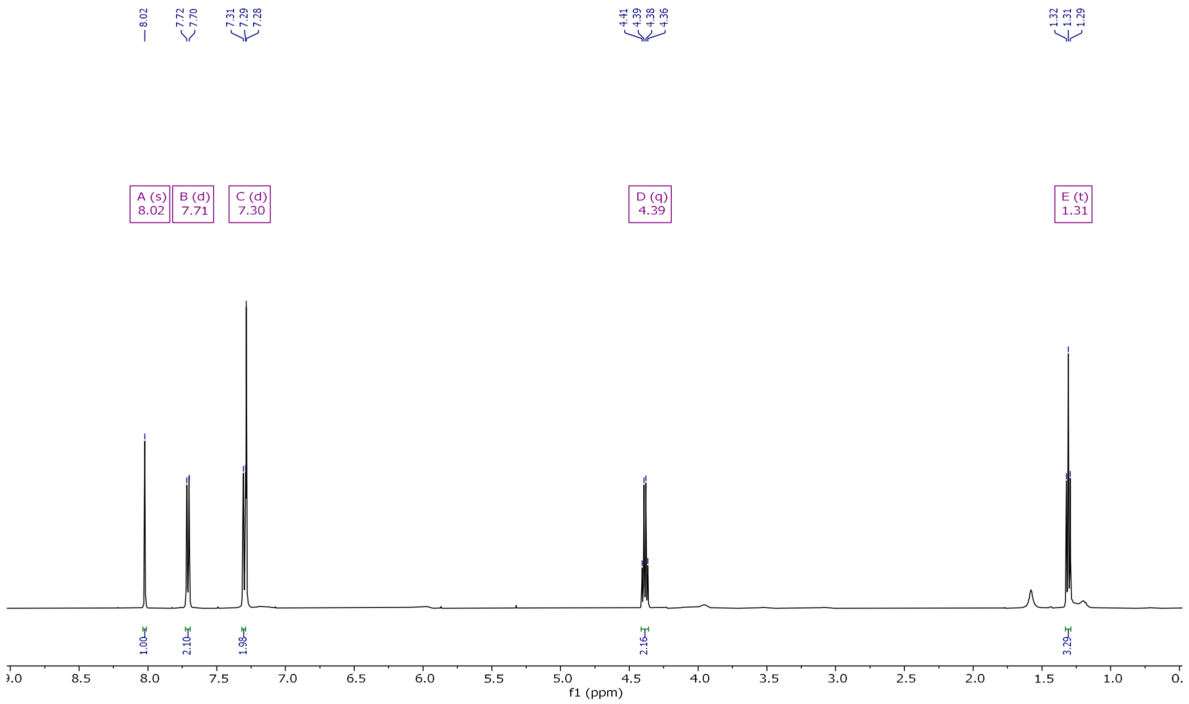

**$^{13}$C-NMR** (126 MHz, $CDCl_3$) of compound 1p

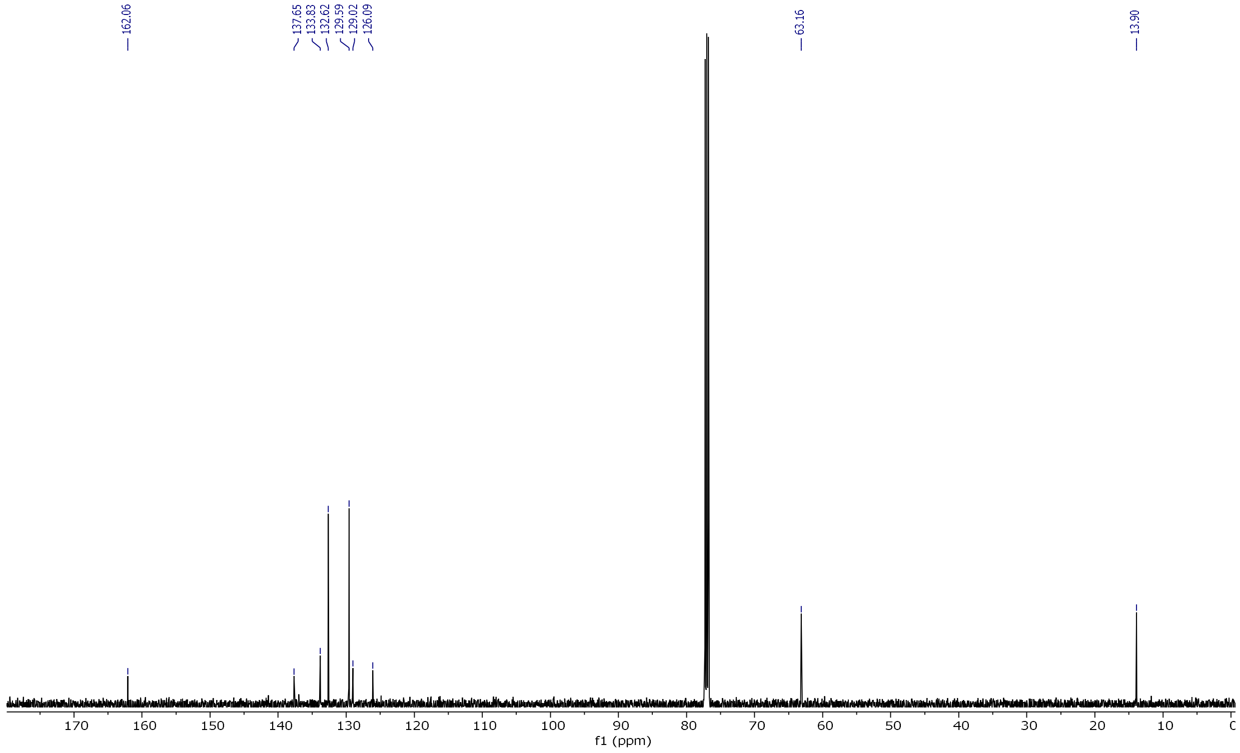

$^{1}$**H-NMR** (500 MHz, $CDCl_3$) of compound 1r

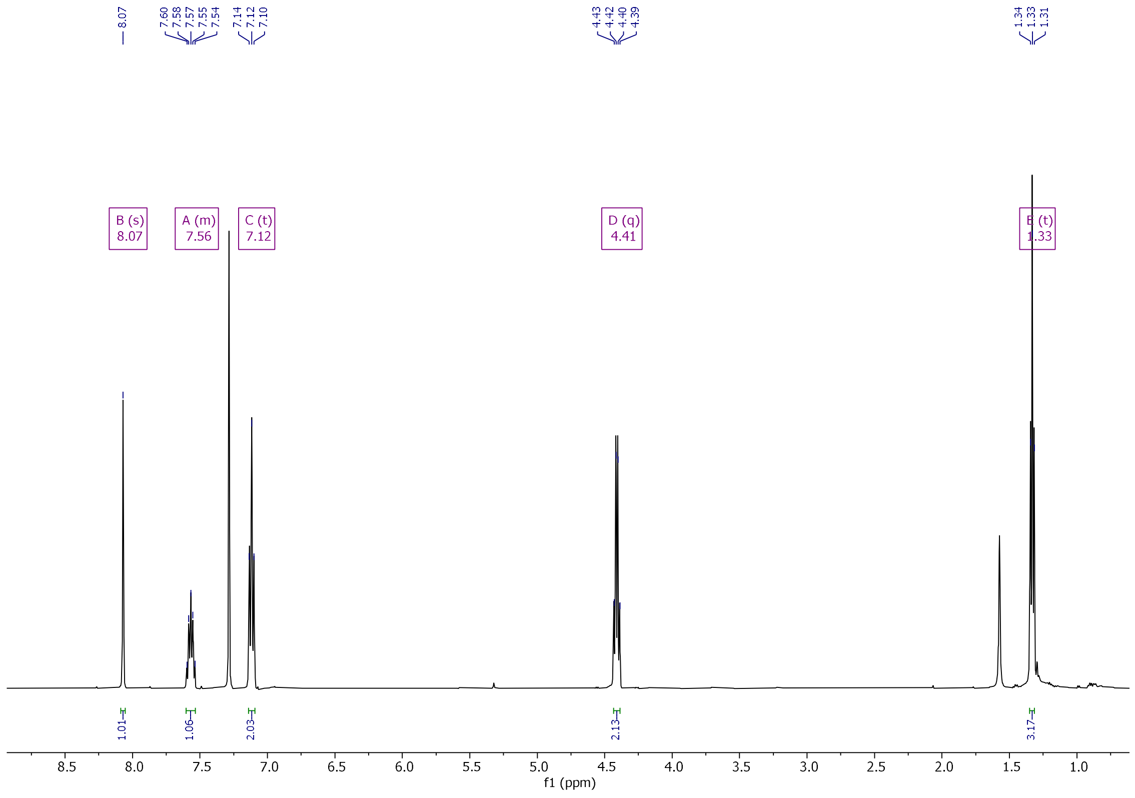

$^{13}$**C-NMR** (126 MHz, $CDCl_3$) of compound 1r

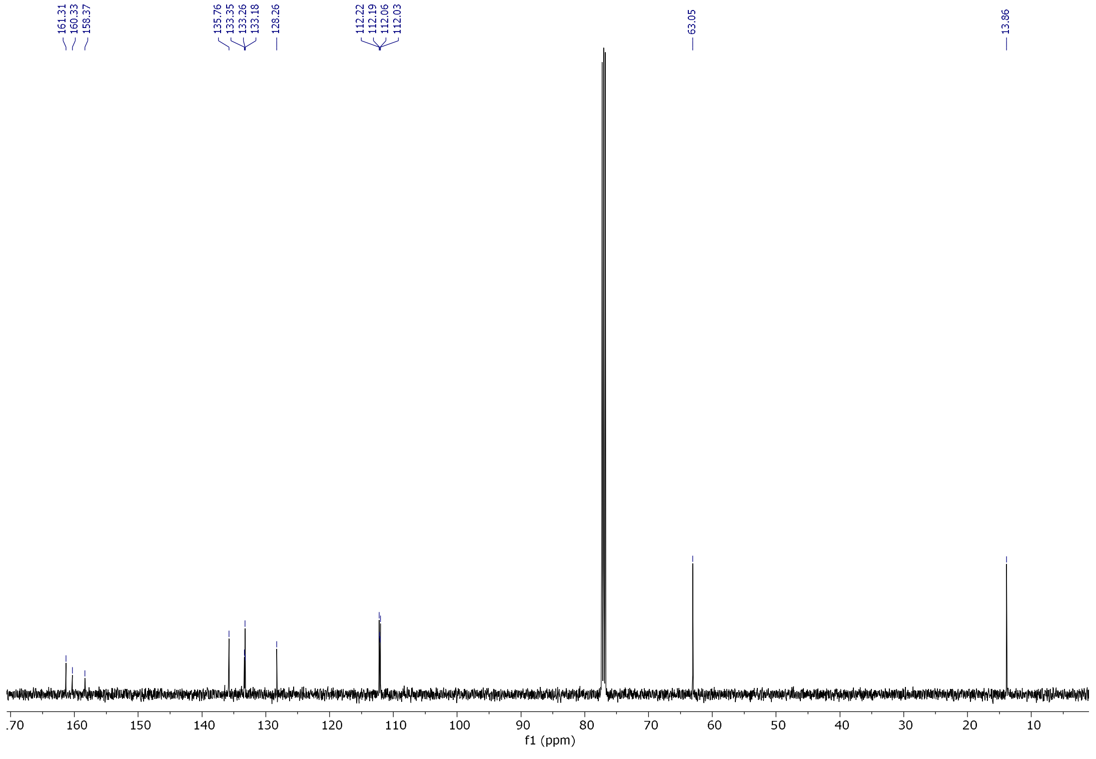

**Series 2**

**$^1$H NMR** (500 MHz, $CDCl_3$) Compound 2d

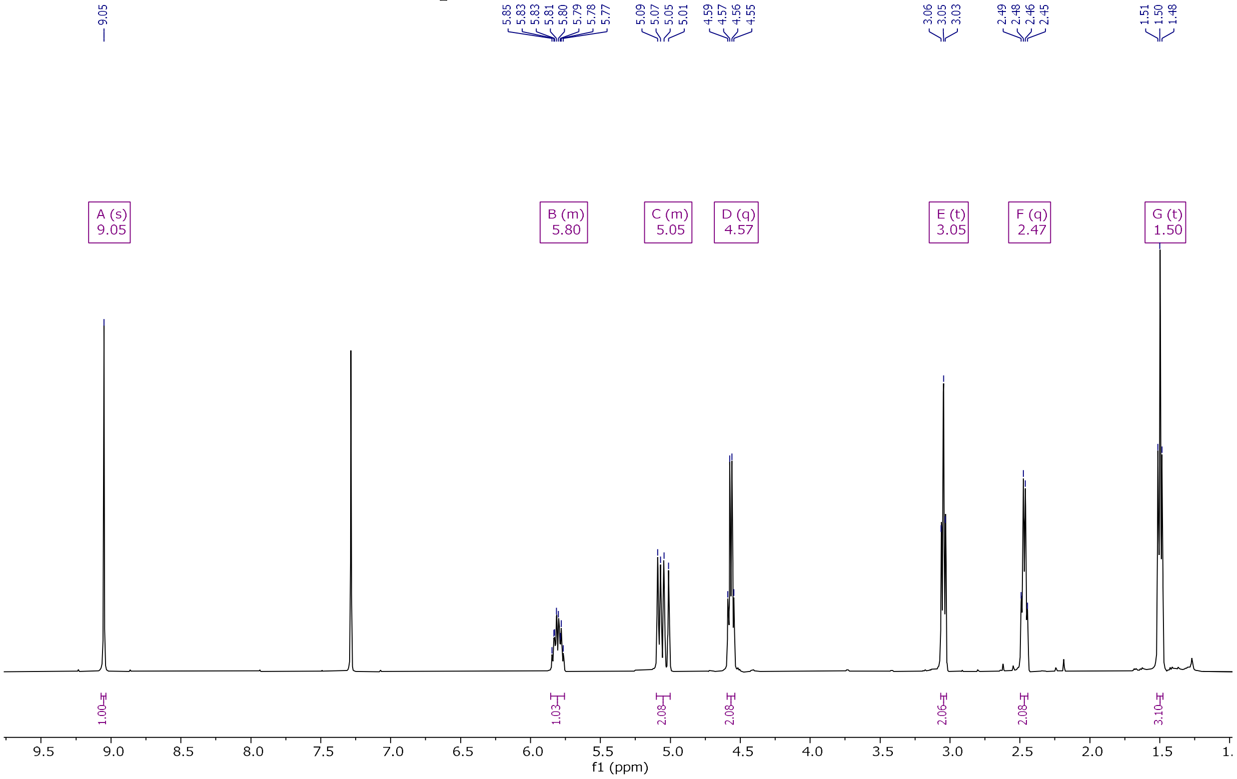

**$^{13}$C NMR** (126 MHz, $CDCl_3$) compound 2d

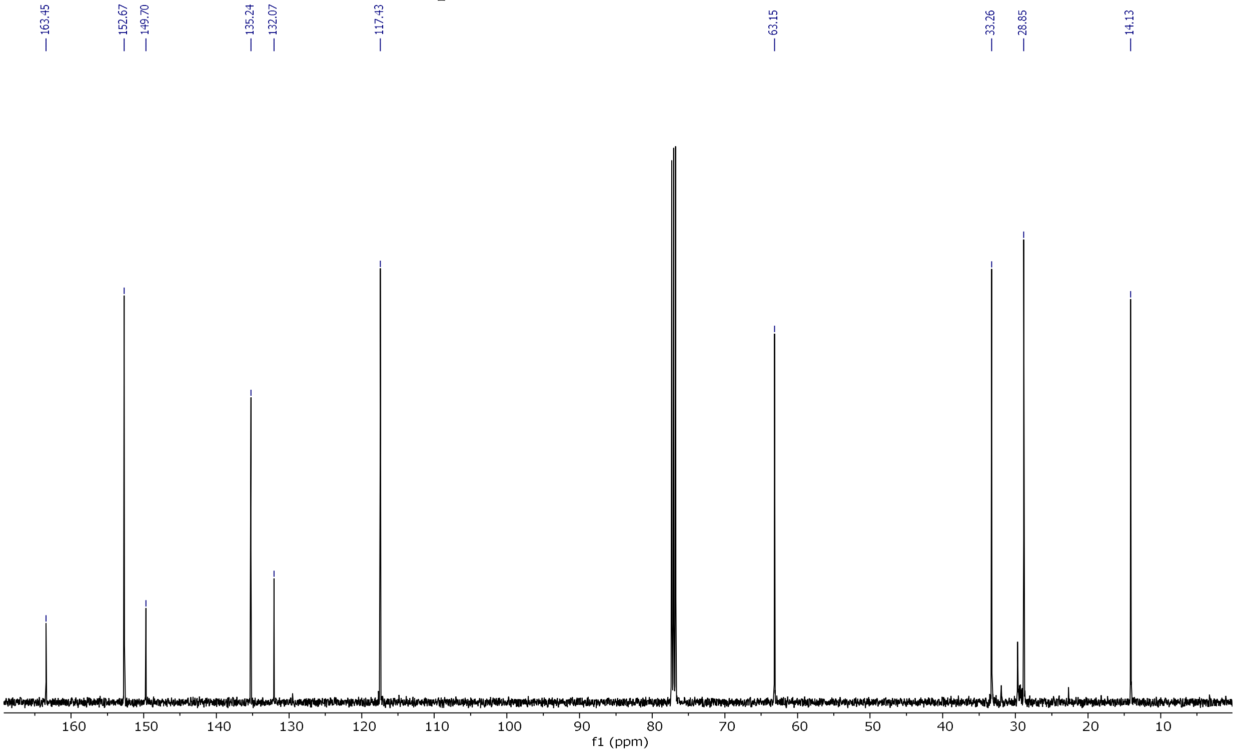

**¹H NMR** (500 MHz, $CDCl_3$) Compound 2r

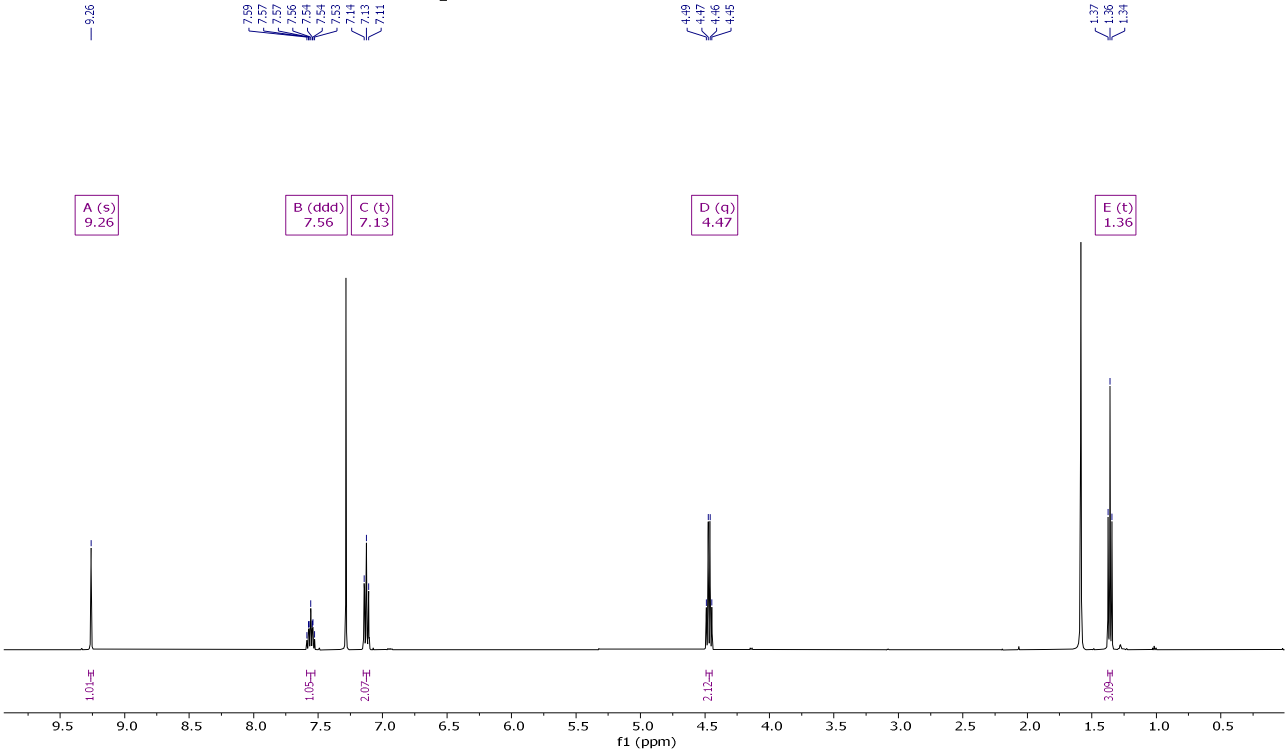


**¹³C NMR** (126 MHz, $CDCl_3$) compound 2r

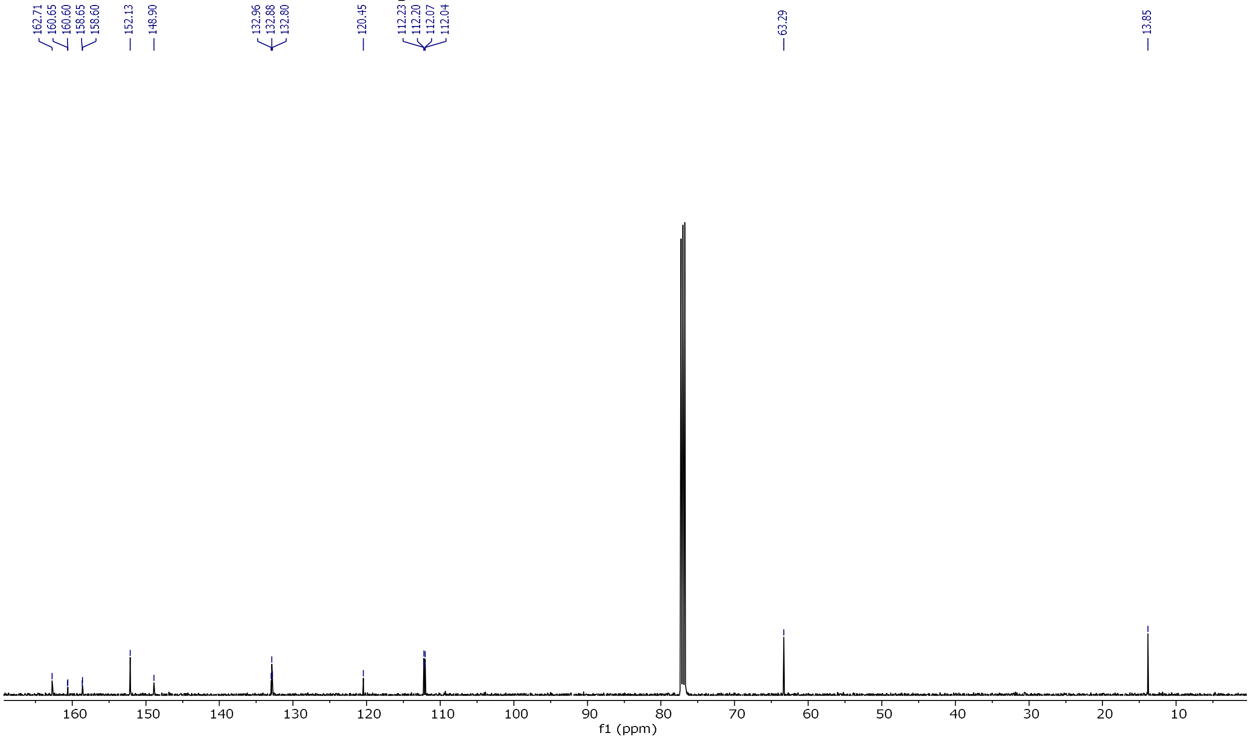

**$^{1}$H NMR** (500 MHz, $CDCl_3$) Compound 2s

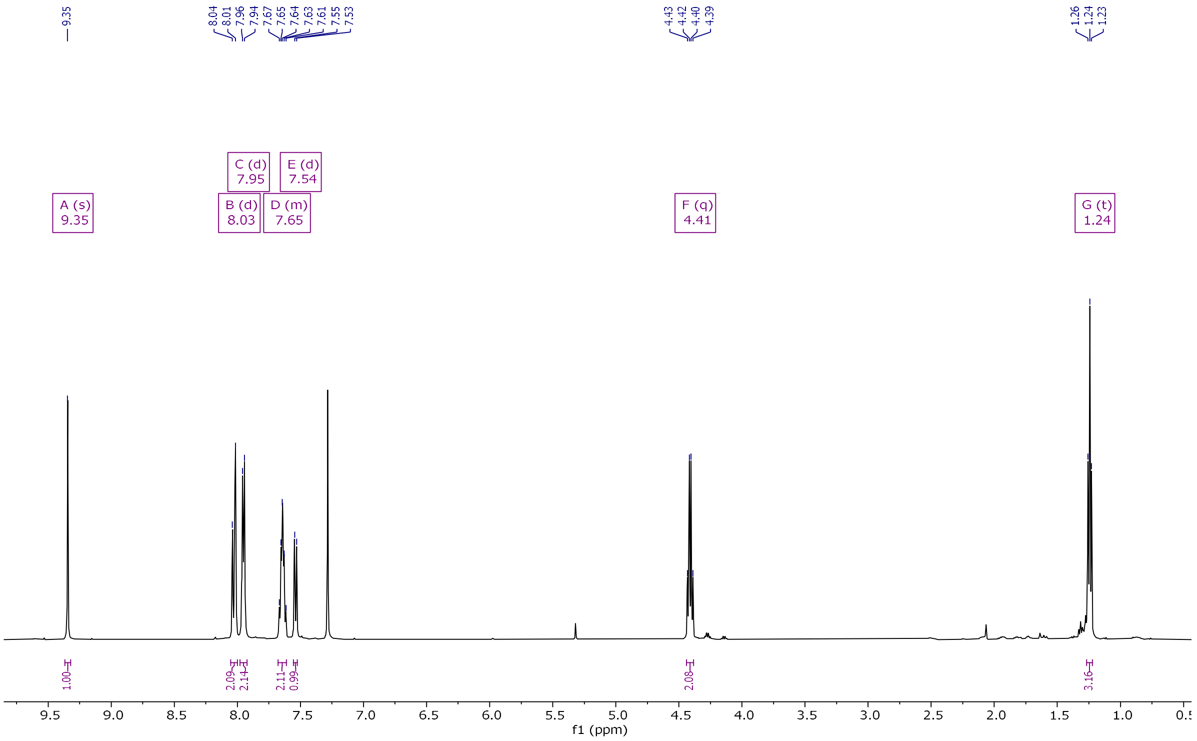


**$^{13}$C NMR** (126 MHz, $CDCl_3$) compound 2s

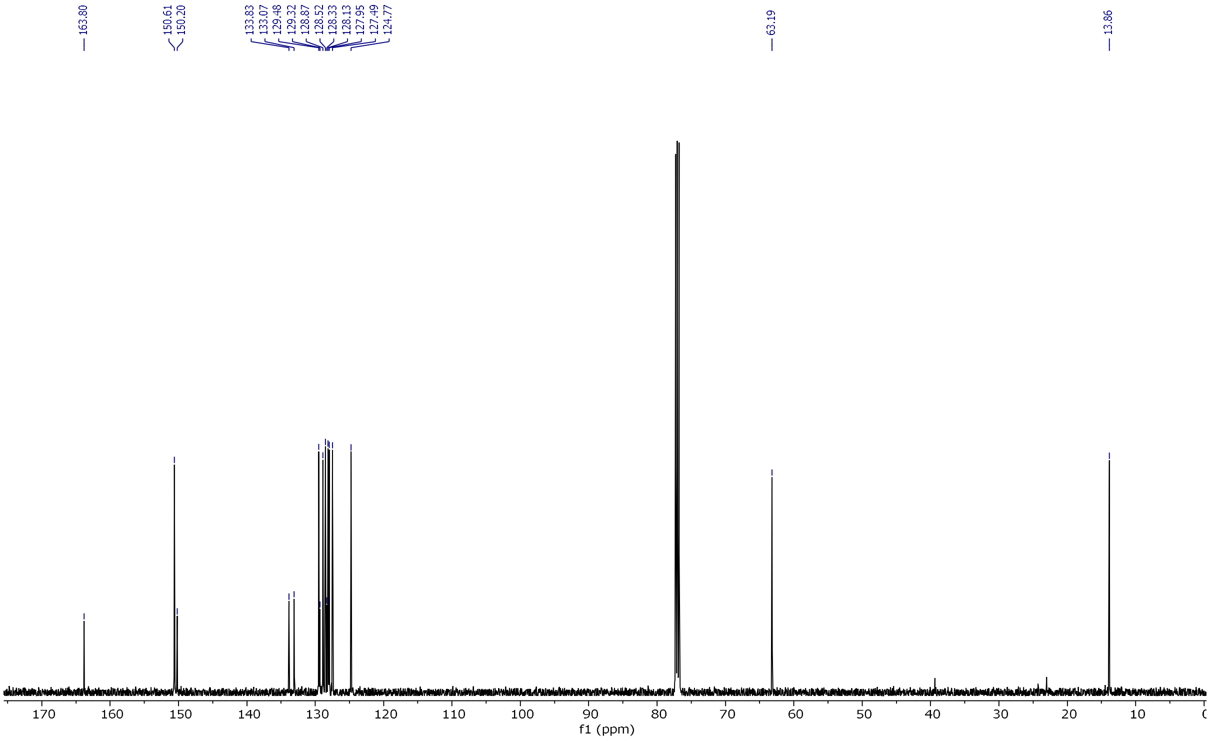

## Series 3

**$^1$H NMR** (500 MHz, $CDCl_3$) Compound 3b

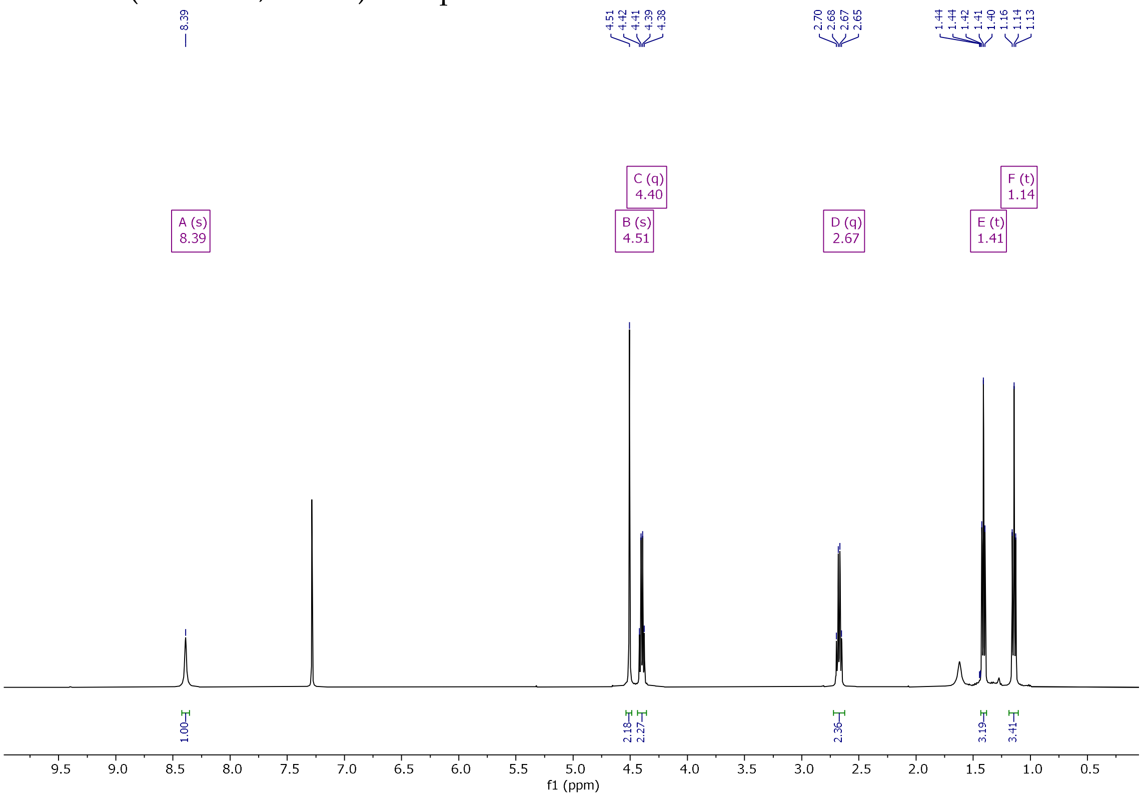


**$^{13}$C NMR** (126 MHz, $CDCl_3$) compound 3b

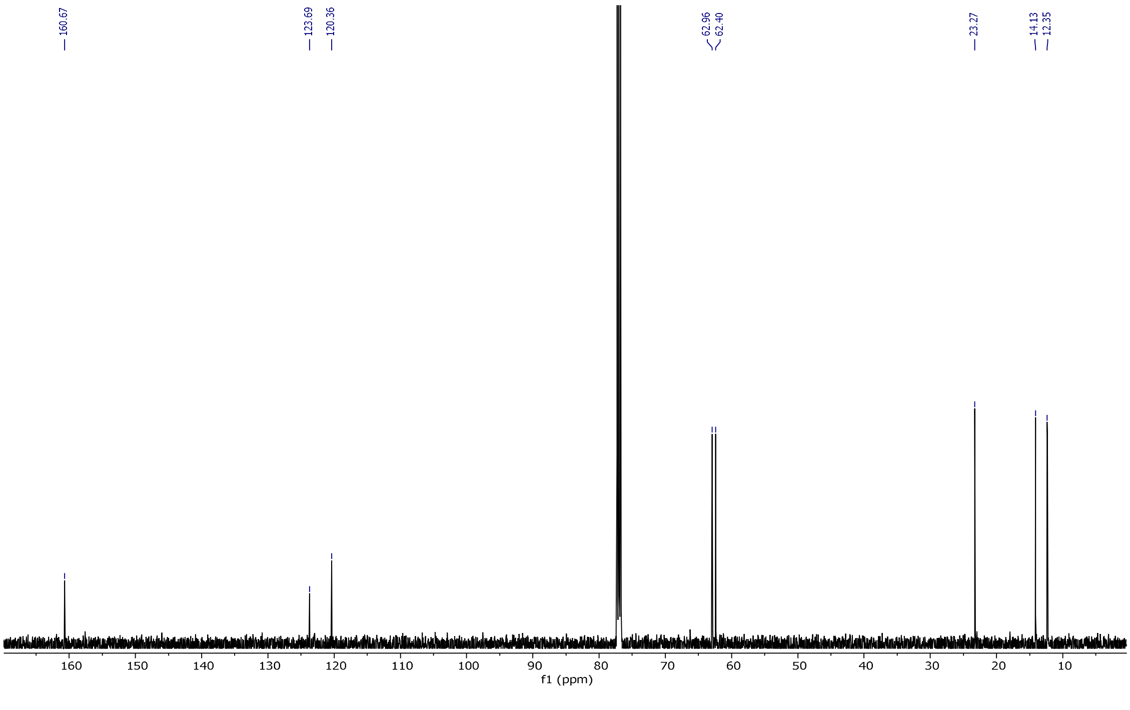

**$^1$H NMR** (500 MHz, $CDCl_3$) Compound 3d

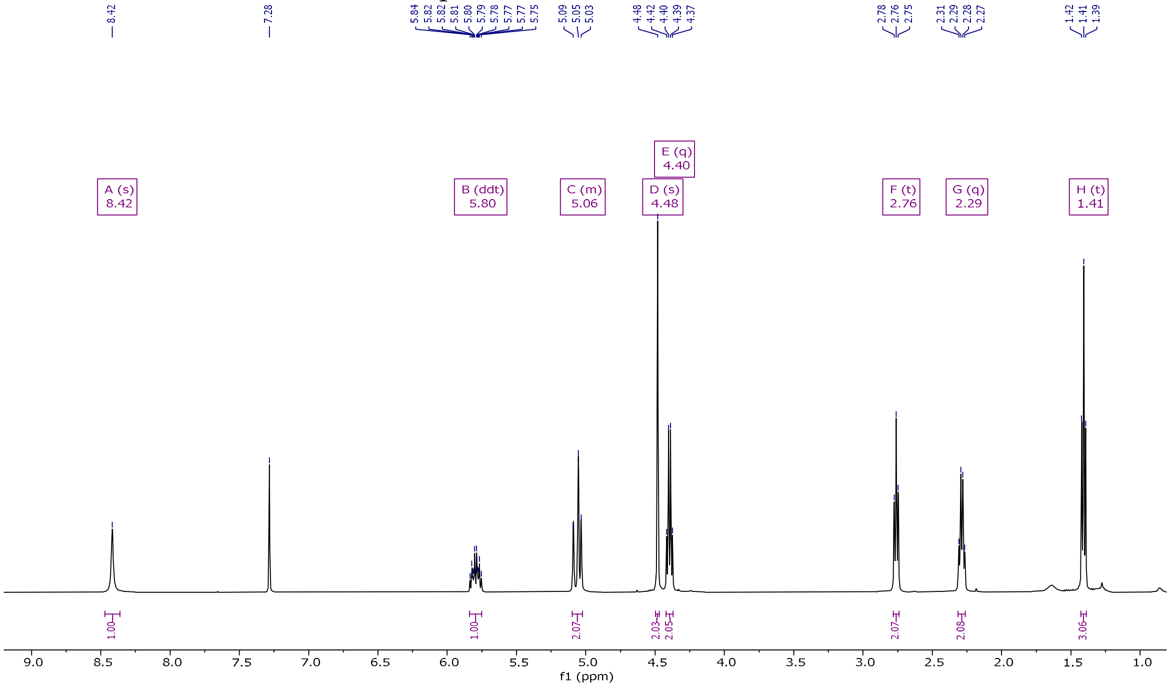


**$^{13}$C NMR** (126 MHz, $CDCl_3$) compound 3d

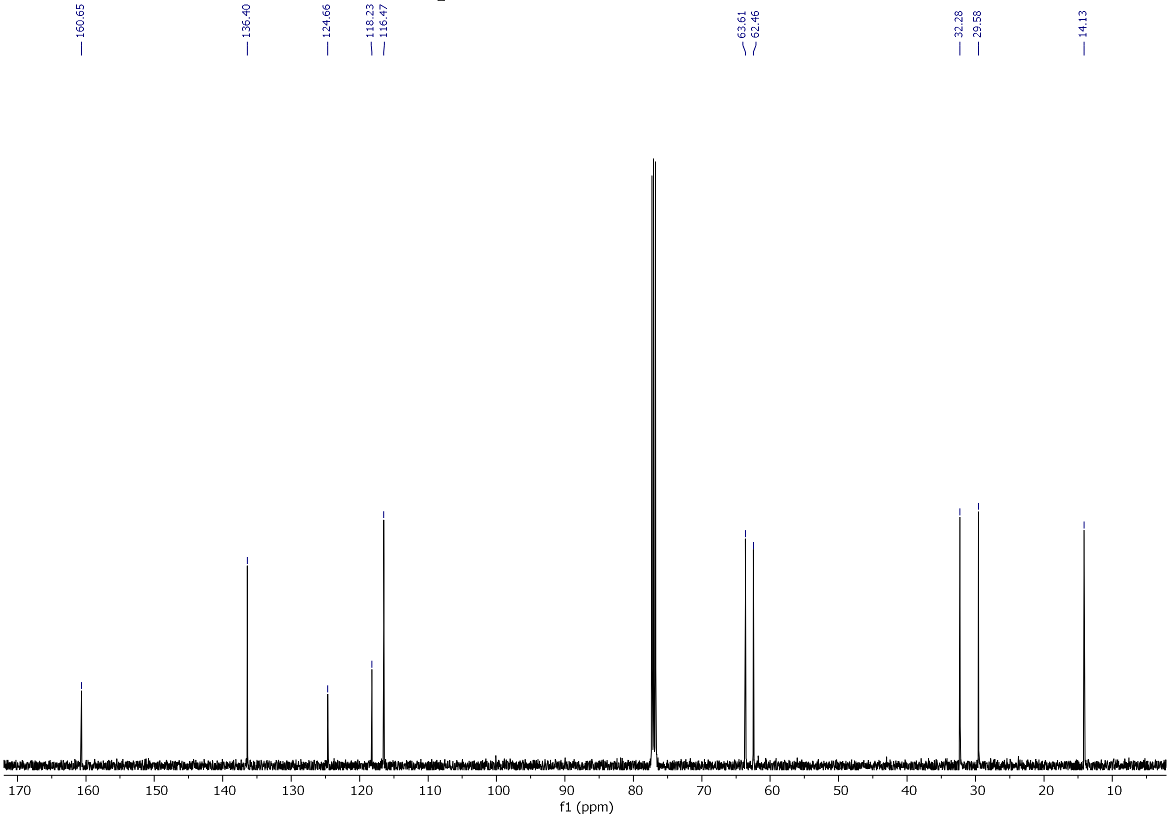

**$^1$H NMR** (500 MHz, $CDCl_3$) Compound 3p

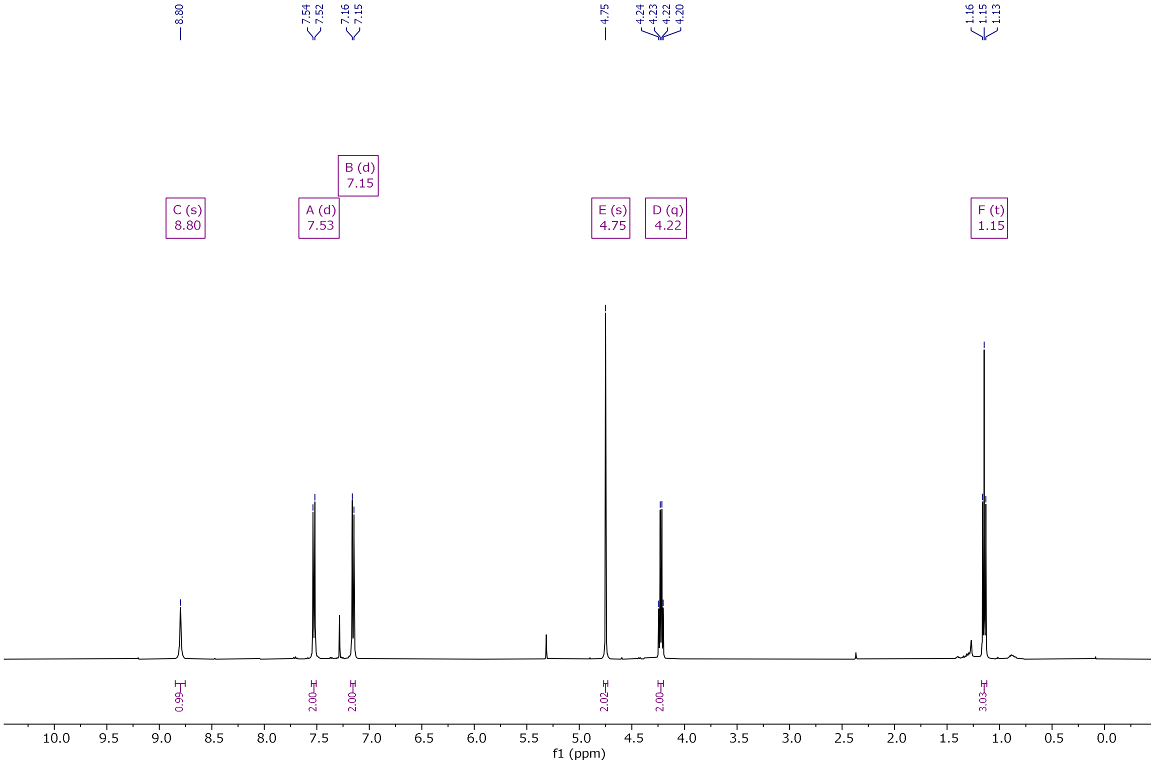


**$^{13}$C NMR** (126 MHz, $CDCl_3$) compound 3p

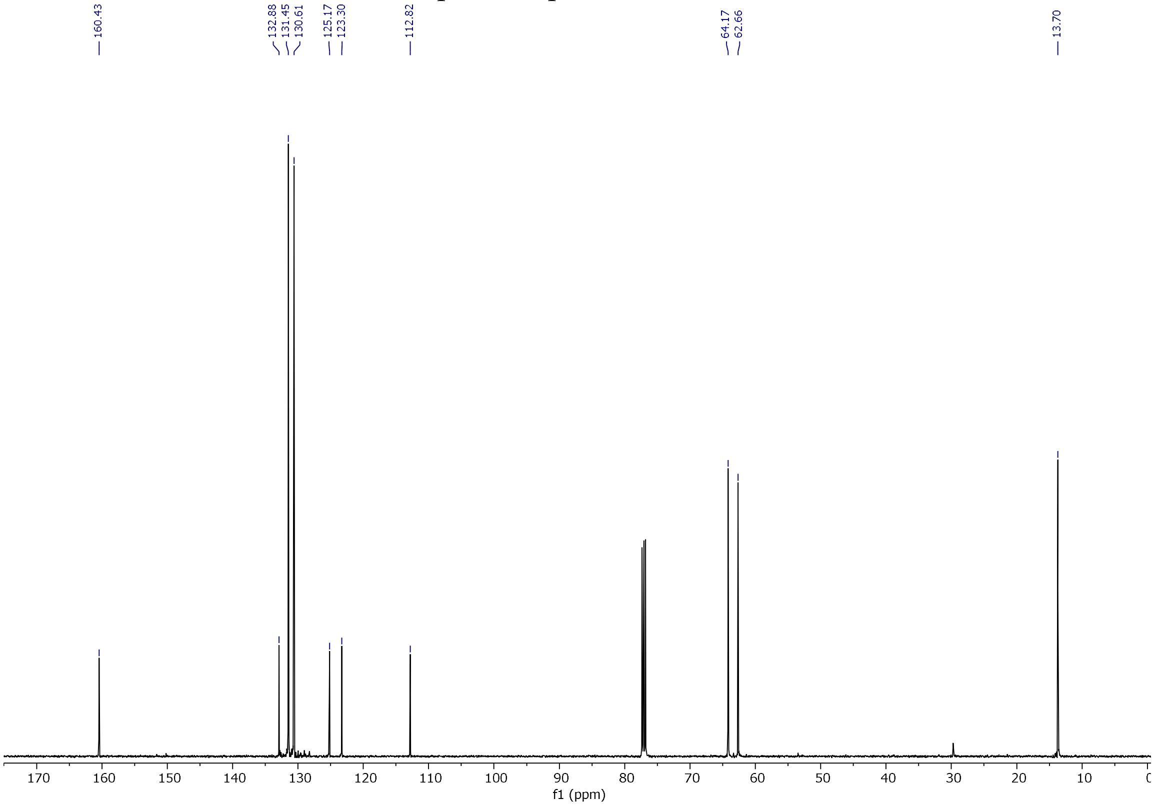

**¹H NMR** (500 MHz, $CDCl_3$) Compound 3r

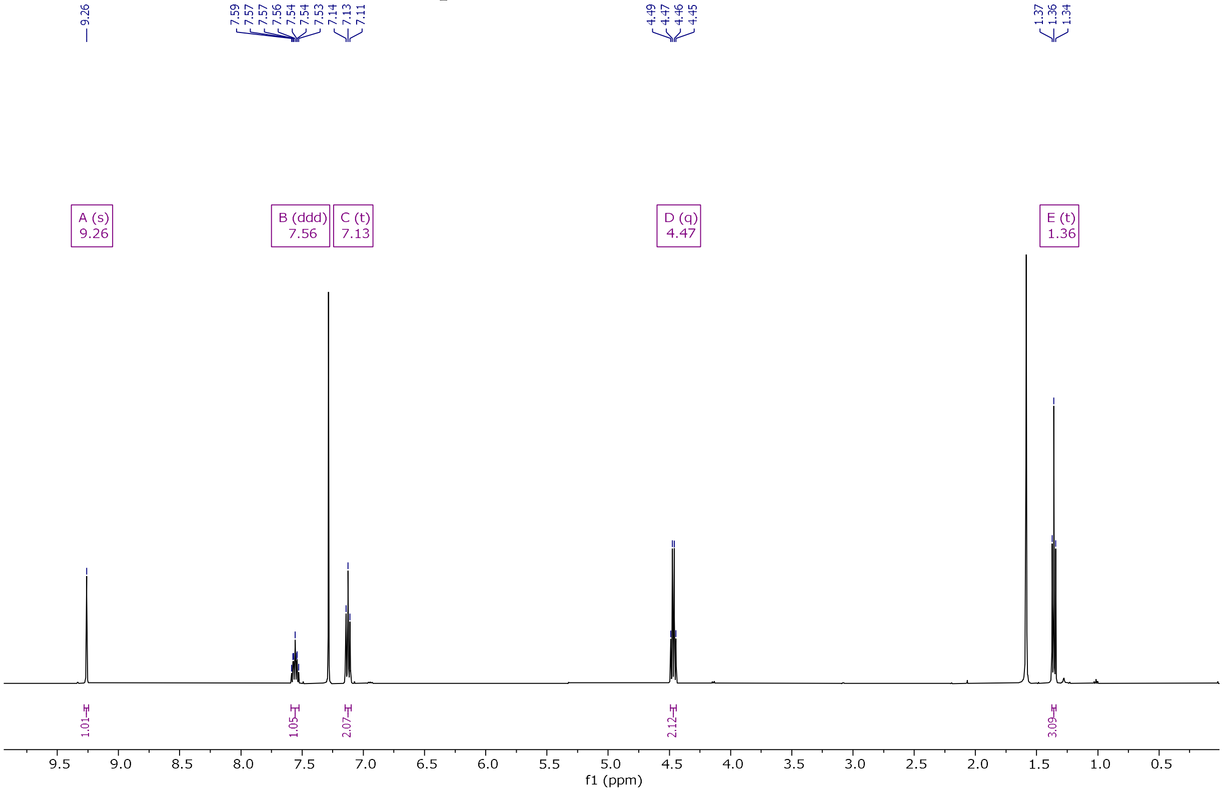

**¹³C NMR** (126 MHz, $CDCl_3$) compound 3r

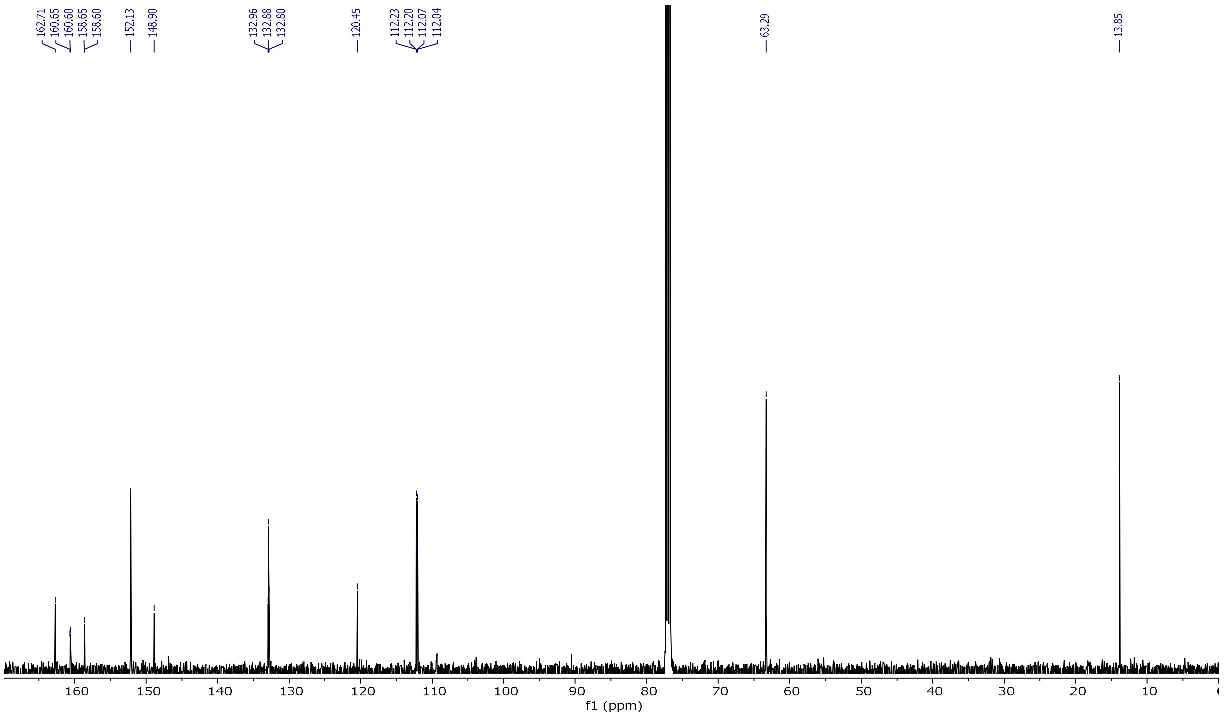

## 4. *M. tuberculosis* gyrase supercoiling inhibition

### 4.1 Inhibition assay

**Table S1**. Inhibition of M. tuberculosis DNA Gyrase activity

| Compound | Structure | Assay IC50 (µM) | | |
|---|---|---|---|---|
| | | **1** | **2** | **Average** |
| 1j | 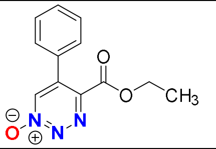 | NI | NI | NI |
| 1k | 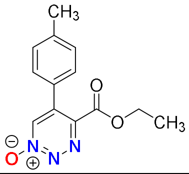 | NI | NI | NI |
| 1m | 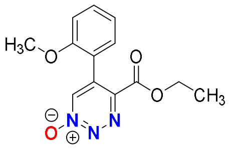 | NI | NI | NI |
| 1o | 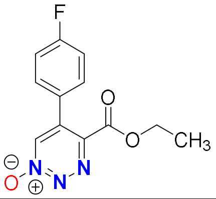 | NI | NI | NI |
| 2j | 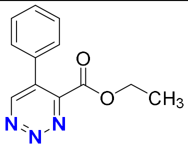 | NI | NI | NI |
| 2k | 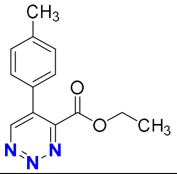 | NI | NI | NI |
| 2o | 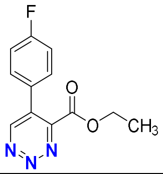 | NI | NI | NI |
| 2n | 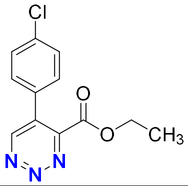 | NI | NI | NI |

| | | | | |
|---|---|---|---|---|
| 3k | 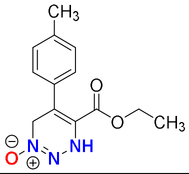 | NI | NI | NI |
| 3n | 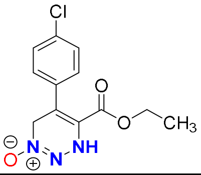 | NI | NI | NI |
| 3o | 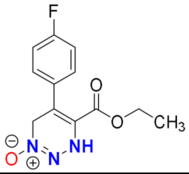 | NI | NI | NI |
| 3s | 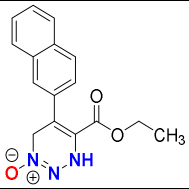 | NI | NI | NI |
| **Moxifloxacin** | 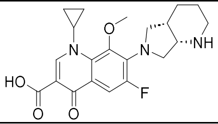 | | | |

*NI= Not Inhibited

## *4.2 Test of compounds*

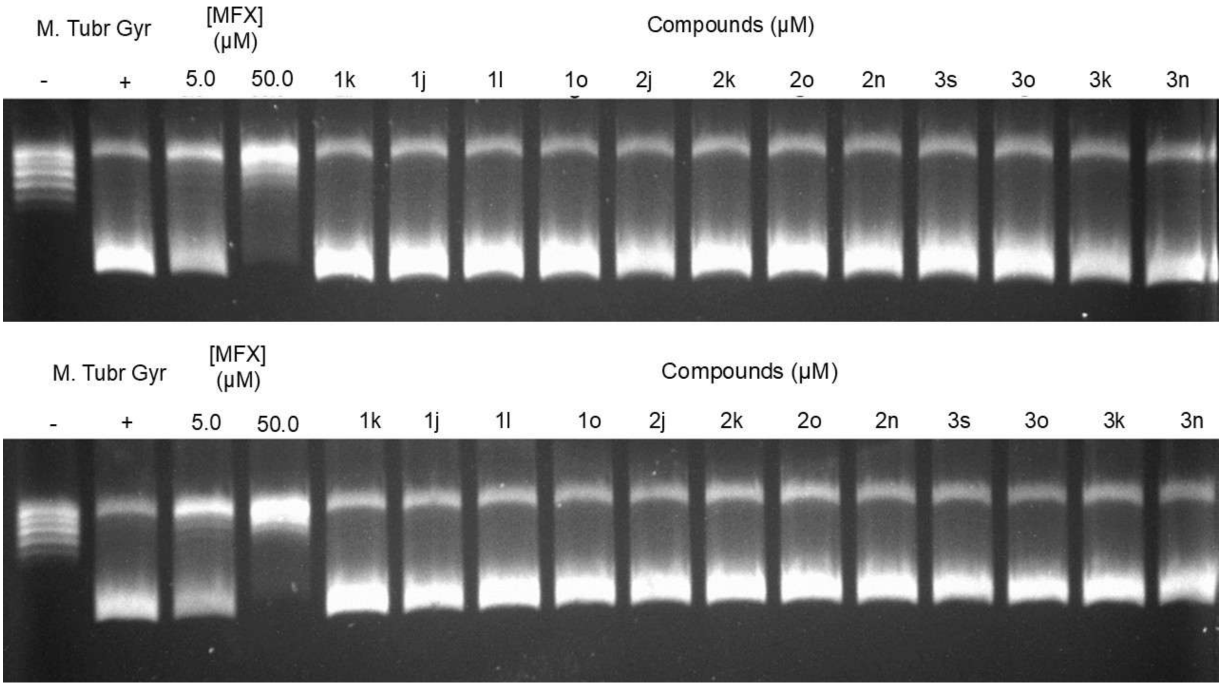


**Figure S2.** Inhibition M. tuberculosis DNA gyrase supercoiling activity by 1,2,3-triazine derivatives at 50 μM. 1) Assay 1; 2) Assay 2

*a. Test of control*

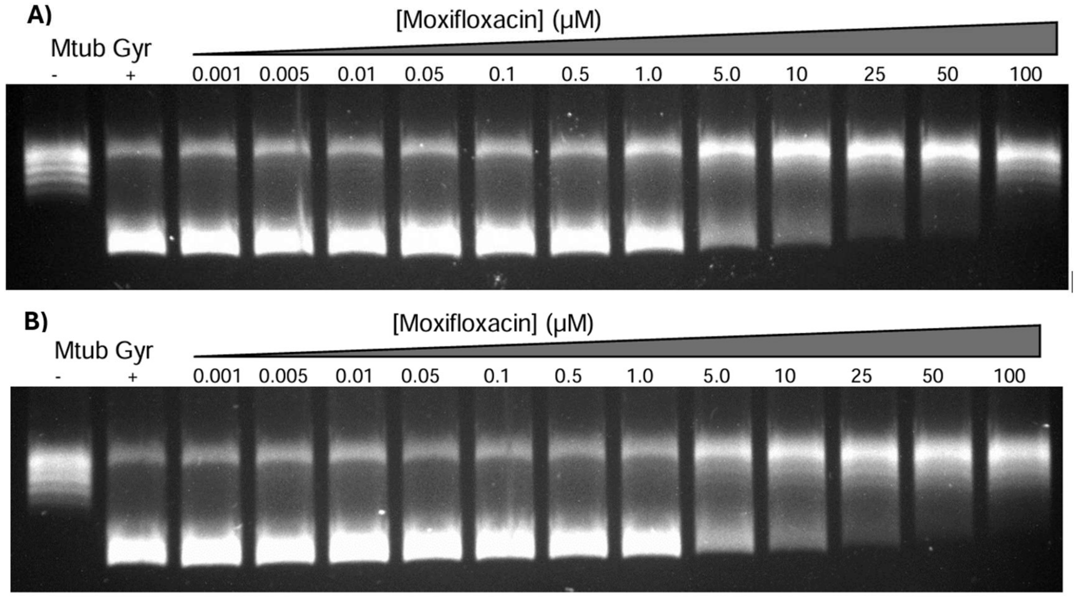


**Figure S3.** Determination of the $IC_{50}$ of moxifloxacin as a control. A) Assay 1 (6.16 μM); B) Assay 2 (5.12 μM)

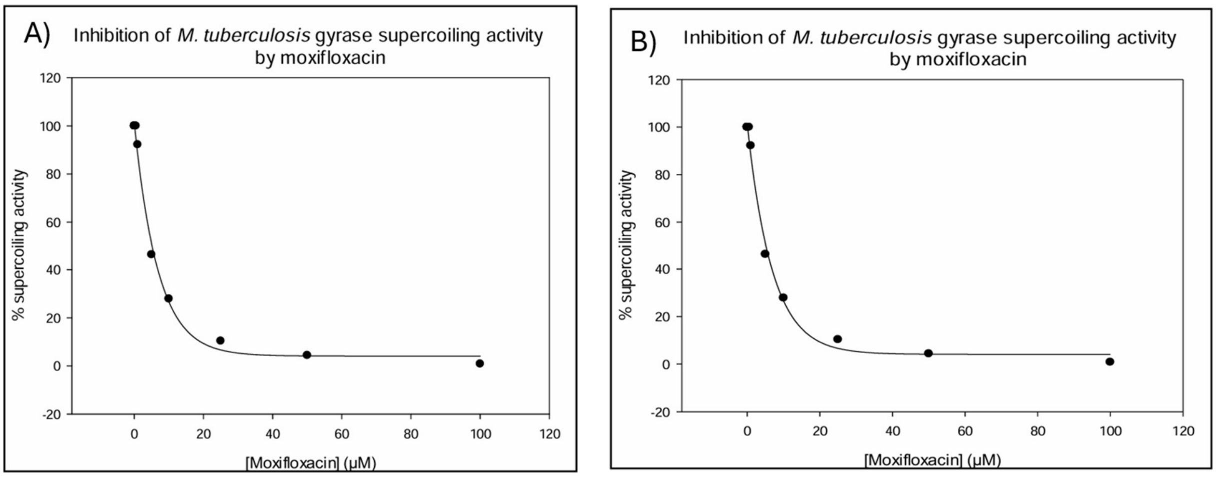


**Figure S4**. Percentage of supercoiling activity by moxifloxacin. A) Assay 1 (6.16 μM); B) Assay 2 (5.12 μM)

5. **References**


a) De Angelis, L.; Zheng, H.; Perz, M.T.; Arman, H.; Doyle, M.P. (2021) Intermolecular [5+1]-Cycloaddition between Vinyl Diazo Compounds and tert-Butyl Nitrite to 1,2,3-triazine 1-Oxide and Their Further Transformation to Isoxazole. Org. Lett. 23, 6542-6546.

b) Rivera, G., De Angelis, L., Al-Sayyed, A., Biswas, S., Arman, H., & Doyle, M. P. (2022). Synthesis of 1, 2, 3-Triazine Derivatives by Deoxygenation of 1, 2, 3-Triazine 1-Oxides. Organic Letters, 24(36), 6543-6547.

c) De Angelis, L.; Haug, G.; Rivera, G.; Biswas, S.; Al-Sayyed, A.; Arman, H.; Larionov, O.; Doyle, M. P. (2023) "Site Reversal in Nucleophilic Addition to 1,2,3-Triazine 1-Oxides" J. Am. Chem. Soc. 145 ,13059-13068.


Ref. [19,20,21] are cited in the supplementary materials.
